# Pose Compensation Method for Robotic Manipulators Based on Transformer

**DOI:** 10.3390/s26175402

**Published:** 2026-08-26

**Authors:** Qingqing Ji, Yuqian Li, Yaxuan Liu, Zhaoxin Li, Min Shi, Dengming Zhu, Zhaoqi Wang

**Affiliations:** 1 University of Chinese Academy of Sciences, Beijing 100049, China; jiqingqing20b@ict.ac.cn; 2 Institute of Computing Technology, Chinese Academy of Sciences, Beijing 100190, China; zqwang@ict.ac.cn; 3 School of Cyber Science and Engineering, University of International Relations, Beijing 100191, China; weidengxie@gmail.com; 4 School of Mathematics Statistics and Mechanics, Beijing University of Technology, Beijing 100124, China; liuyaxuan@emails.bjut.edu.cn; 5 Agricultural Information Institute, Chinese Academy of Agricultural Sciences, Beijing 100081, China; cszli@hotmail.com; 6 Key Laboratory of Agricultural Big Data, Ministry of Agriculture and Rural Affairs, Beijing 100081, China; 7 School of Control and Computer Engineering, North China Electric Power University, Beijing 102206, China; shi_min@ncepu.edu.cn; 8 Taicang-ZK Institute of Information and Technology, Taicang 215400, China

**Keywords:** robotic arm, pose error compensation, Transformer, industrial robot

## Abstract

Industrial robots, particularly six-axis serial manipulators, have been widely deployed in manufacturing workflows including assembly, welding, material handling, inspection and precision machining. As the demand for higher end-effector positioning accuracy and trajectory tracking performance grows, end-position errors induced during manipulator operation—stemming from geometric deviations, joint friction, load fluctuations, current surges, as well as variations in velocity and acceleration—have emerged as a critical bottleneck limiting high-precision applications. Conventional error compensation approaches mostly rely on geometric calibration, empirical formulas or fixed regression algorithms, which struggle to adequately characterize error trends featuring strong temporal dependencies, nonlinearity and multi-factor coupling. To address the aforementioned limitations, this paper takes the UR5 industrial manipulator as the research object. Leveraging the NIST-released dataset for manipulator positional accuracy degradation monitoring, this study develops and implements a physics-aware Transformer-based compensation framework that integrates a physics-consistent constraint loss and a nonlinear exponential error amplification strategy with a standard Transformer encoder for end-effector positional accuracy degradation. Multiple variables including target joint position, velocity, acceleration, torque, motor current and control current are selected to construct time-window input vectors, which are used to train the Transformer regression model to capture the correlation between historical motion states and real-time end-effector positional accuracy degradation. Experimental results demonstrate that the proposed Transformer model can fully capture temporal contextual correlations and multi-feature fusion information embedded within manipulator kinematic data, delivering superior error compensation performance for the six-dimensional end-effector pose error prediction task. The self-attention-based time-series modeling framework is well-suited to the nonlinear, coupled and time-varying characteristics of manipulator operational errors. This work provides valuable references for accuracy enhancement of industrial robots and the design of intelligent error compensation schemes. This work provides valuable references for accuracy enhancement of industrial robots and the design of intelligent error compensation schemes, with the proposed physics-aware strategies being model-agnostic and potentially extensible to other regression architectures.

## 1. Introduction

Industrial robots are essential execution parts in intelligent manufacturing systems. They can perform high-precision tasks in harsh production environments and replace human beings in dangerous work. Compared with traditional fixed mechanical equipment, six-axis robotic arms have two advantages. They are more flexible and easier to program and are widely applied in fields such as automobile manufacturing, electronic product assembly and automated production. As industrial production is trending towards more refinement and intelligence, the actual pose accuracy of robotic arm’s end-effectors is more and more important [1]. It not only directly affects the processing quality of working procedures but also influences the stability and quality consistency of the entire production line [2].

In the process of actual operation, the motion trajectory of robotic arms can hardly fully meet the theoretical design standards, nor accurately reach the position and posture commanded by the controller. There are many factors contributing to the end pose errors including geometric errors and non-geometric errors. Geometric errors include connecting rod dimension errors, joint zero offset errors and installation errors. Non-geometric errors include joint friction, transmission gaps, elastic deformation, temperature changes, end load changes and control current fluctuations [3]. For serial robotic arms such as UR5, tiny deviations in joint space will be amplified along the motion chain, which can result in amplification or coupling effect in Cartesian space, which will in turn lead to all-round, nonlinear and time-varying end pose errors.

Traditional accuracy improvement methods for robotic arms mainly include geometric parameter calibration, linear regression compensation and dynamic model-based compensation. These methods achieved a certain level of good performance under stable working conditions, but they have many downsides, like requiring complicated modeling and having strict requirements on working environments and operating conditions. Simultaneously, they have poor performance in modeling and adaptation when dealing with non-geometric errors caused by load changes, moving speed, temperature and operating history.

With the continuous accumulation of operating data from industrial robot controllers, machine learning methods, especially time-series modeling methods, provide new ideas for error compensation. By making use of the data-driven models, machines can learn error variation rules from historical operating data, and this method has gradually become the mainstream method for robotic arm error prediction and compensation [4].

Pose error compensation for robotic arms is a technical method based on error modeling and error prediction. By adjusting the robot’s target position, kinematic parameters or control commands, we can minimize the deviation between the actual pose and the theoretical pose of the end effector. Compared with traditional pose calibration methods, error compensation focuses more on reducing final errors and improving the overall accuracy of the completion of tasks.

According to different compensation objects and modeling approaches, existing robotic arm error compensation methods can be divided into kinematic parameter calibration compensation, compliance modeling compensation, and machine learning-based data-driven compensation. For parameter model-based compensation, there are two ways to improve the end pose precision after identifying robotic kinematic parameters. The first way is to import calculated parameters into the robot control system. The second one is to correct forward and inverse kinematic calculations. This method has two advantages, which are clearer physical significance and a higher performance when geometric errors dominate the overall deviation [5]. However, it has limited capability in modeling and eliminating non-geometric errors and dynamic errors caused by external working conditions.

Kinematic parameter calibration compensation is the most traditional and widely adopted method for robotic arm pose error compensation. Normally based on the robot kinematic model, this method establishes the relationship between end pose error and kinematic parameter deviation by measuring the actual end-effector pose under multiple joint configurations and comparing it with theoretical pose calculated by the kinematic model.

Then, algorithms such as the least square method, weighted least square method, Levenberg–Marquardt algorithm, extended Kalman filter and intelligent optimization algorithms are applied to identify geometric parameter errors, including link length error, link torsion angle error, joint zero offset and joint bias. The calibrated parameters are used to revise the controller model or offline motion trajectory of the robotic arm.

This method has many advantages. It has clear physical significance and can well eliminate end errors caused by geometric factors, such as manufacturing deviation, assembly error and joint axis deflection. It plays a vital role in improving the absolute positioning accuracy of industrial robots dominated by geometric errors [6]. Common kinematic modeling methods include the standard D-H model, modified D-H model, CPC model, POE model and dual quaternion model. These models differ in parameter continuity, completeness, minimization and practical implementation complexity.

Overall, kinematic parameter calibration is suitable to serve as a fundamental method for robotic arm error compensation [7]. However, it has downsides as well. The compensation performance highly depends on the accuracy of the geometric model. When robotic arm errors are caused by non-geometric factors such as load variation, joint flexibility, friction, temperature drift and dynamic control errors, pure kinematic calibration cannot completely eliminate residual end errors. Therefore, in complex industrial situations, kinematic parameter calibration usually needs to be used with other non-geometric error compensation methods.

Compliance modeling compensation is an important research direction for solving non-geometric errors of robotic arms [8]. Industrial robotic arms usually adopt a series of cantilever structures. Although they are regarded as rigid body systems in theoretical modeling, their links, joints, reducers, bearings and transmission chains will produce tiny elastic deformations under the effect of self-weight, end load, contact force and machining force during actual operation [9]. These local deformations transmit along the motion chain and accumulate at the end, finally causing pose deviations of the end effector.

The core idea of compliance compensation is to establish the mapping relationship among external force, joint torque, structural stiffness and end displacement error. It estimates the elastic deformation of the end effector according to the current robot configuration and force condition and reversely corrects the target pose or joint control commands. Common compliance modeling methods include finite element analysis, matrix structural analysis and virtual joint method. Finite element analysis can accurately describe the local deformation of links and mechanical structures, which is suitable for structural design and high-precision offline analysis. Matrix structural analysis regards links as beam structures and derives local and overall stiffness matrices from the perspective of structural mechanics. The virtual joint method equates the flexibility of joints, transmission parts and links to virtual springs, and adds flexible parameters on the basis of rigid kinematic chains. It is widely used in joint stiffness identification and Cartesian stiffness prediction [10].

Compared with kinematic parameter calibration, compliance compensation can better explain end deviations caused by load changes, self-weight and external forces. It is especially suitable for industrial tasks with obvious force effects, such as drilling, milling, polishing and assembly. However, this method also has limitations. Stiffness parameters will change with robot configuration, load direction, temperature and mechanical wear. Accurate identification of the stiffness matrix often requires additional sensors and experimental equipment. Moreover, a single compliance model cannot fully cover complex errors such as thermal drift, friction hysteresis and dynamic control errors. Therefore, compliance compensation is generally regarded as a key theoretical foundation for non-geometric error compensation research.

With the advancement of machine learning technology, data-driven position error correction for robotic arms has become a popular research topic in recent years [11]. Different from traditional model-based methods, this approach does not rely entirely on accurate physical error models [12]. Instead, it collects massive robot operating data to explore the internal correlation between multiple input variables and end position deviations. The common input variables include joint angle, joint speed, end load, temperature, control current and torque.

Typical machine learning models applied in this field include support vector machine regression, random forest, gradient boosting decision tree, Gaussian process regression, artificial neural network, radial basis function network, recurrent neural network and deep neural network, etc. [13]. The core advantage of machine learning models lies in their powerful nonlinear approximation capability [14]. They can effectively find error compensation solutions even when accurate physical modeling of complex error sources is unavailable.

Among these methods, neural network models show the abilities of self-learning ability, environmental adaptability and a certain level of anti-interference performance. They are particularly suitable for achieving compensation for complex errors caused by the coupling of multiple geometric and non-geometric factors, which cannot be fully solved by traditional calibration and static modeling methods [15].

In deep learning research, feed-forward neural networks are commonly adopted to establish static error mapping relationships [16]. They take the real-time joint position or ideal end-effector pose as input and output the corresponding error compensation value. This method features a simple structure and is easy to train and deploy in practical robotic systems. However, it only focuses on static spatial errors and ignores time-dependent dynamic errors during robot movement [17].

In fact, robotic arm errors are not only determined by the current joint configuration, but also affected by historical motion states, including previous velocity, acceleration, motor current, load variation and thermal conditions. To address such dynamic and cumulative errors, temporal models such as recurrent neural networks (RNNs) [13], Long Short-Term Memory (LSTM) and Transformer have been gradually applied to robotic arm error compensation tasks [18].

Different from static feedforward networks, temporal models can utilize historical state information to predict error variation trends. They show unique advantages in handling time-delay errors, cumulative errors and other time-varying deviations in robot operation. Benefiting from the attention mechanism, temporal data modeling further provides new ideas for dynamic error compensation [19]. Specifically, the Transformer model can capture long-range dependencies among multi-stage operating states and dynamically adjust attention weights according to different task characteristics, making it suitable for modeling the complex nonlinear correlation between multi-dimensional robot operating parameters and end-effector pose errors [20].

Inspired by the research of Chen et al., tree-based models and ensemble learning methods can also be applied to robotic arm error compensation research [9]. The random forest model reduces the over-fitting risk of single decision trees by integrating a large number of tree structures and possesses strong capability in fitting the nonlinear relationships among complex features. Gradient boosting models such as XGBoost and LightGBM iteratively fit residual errors in each training round to improve prediction accuracy, showing prominent anti-interference performance in structured data modeling [21].

Compared with deep neural networks, tree-based models have faster training speed, simpler hyper-parameter optimization and lower sensitivity to feature scales. Therefore, they are suitable for benchmark experiments and feature importance evaluation in error compensation tasks. Nevertheless, tree models are weak in capturing temporal information and require manual extraction of window-based and statistical features to make up for the lack of time-series modeling ability. For robotic arm pose error compensation, tree-based models can serve as reliable contrast models to verify the effectiveness of input features and the necessity of nonlinear error modeling.

In contrast, Yang et al. pointed out that temporal neural networks have stronger modeling advantages when fully utilizing continuous motion information in robot operation [22]. Different from static tree models, temporal neural networks can automatically mine time-dependent error rules from sequential operating data, which is more adaptable to the dynamic and cumulative characteristics of robotic arm pose errors.

In terms of implementation forms, robotic arm error compensation can be realized either by modifying controller parameters or correcting the planned trajectory and target pose. The first method updates the internal kinematic parameters of the robot controller to make the theoretical model match the actual mechanical properties of the robotic arm. In contrast, the second method does not change the underlying controller parameters. Instead, it corrects the target pose based on the predicted errors, so that the actual end position can be closer to the ideal position [23].

For experimental platforms and closed commercial robot systems, target pose compensation is easier to implement and more compatible with external data-driven models [24]. The Transformer-based error prediction and compensation method adopted in this study falls into this category. It utilizes historical motion states and multi-modal sensing data to predict real-time end pose deviations and complete online compensation [14]. This data-driven method does not require explicit modeling of every error source. It only learns potential error variation rules from massive operating data. Therefore, it is well-suited for solving complex coupled errors caused by the superposition of current fluctuation, moving speed, acceleration, variable load and other dynamic factors during robotic arm operation [25].

Despite the continuous progress of robotic arm pose error compensation technology, certain limitations still remain in practical applications. Data-driven compensation methods rely heavily on the quality and coverage of training data. If the training dataset fails to fully reflect the varying working conditions in real industrial scenarios, the generalization ability of the prediction model will be greatly restricted [26]. Moreover, error compensation systems need to balance prediction accuracy and real-time performance [27]. Although large-scale deep network models can achieve high prediction accuracy, their complex structural computation may cause time delays and cannot fully meet the high real-time requirements of robot dynamic control [28].

The final compensation effect is determined not only by the model’s prediction capability, but also by the coordination of transformation accuracy, pose representation rationality, measurement noise and the execution precision of the servo system. Although pure black-box data-driven models can improve compensation results, they lack explicit analysis of error sources and physical interpretability. Such methods cannot satisfy the high standards of interpretability and stability required in reliable industrial production. Therefore, future research should combine kinematic models, multi-sensor information and intelligent learning algorithms to construct an integrated compensation scheme that achieves high accuracy, strong generalization and reliable practical deploy ability [26].

The existing literature indicates that robotic arm pose calibration and error compensation are not independent research topics but form an integrated procedure for robot accuracy improvement. Pose calibration provides basic kinematic models and parameter correction foundations, while error compensation further eliminates residual errors and time-varying deviations that remain after calibration. Traditional model-based compensation methods possess definite physical significance and stable performance under regular working conditions. However, they show limited adaptability when handling complex, variable and highly nonlinear error characteristics in actual industrial scenarios. In contrast, data-driven methods can effectively fit complex nonlinear mapping relationships between robot operating states and pose errors, yet they rely heavily on sufficient and high-quality training data and still face inherent limitations in generalization ability and model interpretability.

Against the above research background, this paper focuses on the end pose error of the UR5 robotic arm and conducts in-depth research on data-driven error compensation strategies. This study adopts a multi-feature time-series modeling method for robotic arm error prediction. It integrates multiple real-time operating features, including joint position, motion velocity and control current, and applies the Transformer model to capture continuous error variation trends within consecutive time periods. This research provides a feasible and effective solution for dynamic pose error prediction and compensation of industrial robotic arms.

This study focuses on the end pose error prediction and compensation of the UR5 robotic arm. The main research contents include dataset integration, error label generation, model construction, experimental evaluation and systematic design. Firstly, based on the public NIST robotic arm position accuracy degradation dataset, this study reads and cleans the original CSV files collected under various working conditions, including different loads, moving speeds and cold start states. Key features such as joint current and joint velocity are extracted to analyze the correlation between robot control variables and end pose errors.

Subsequently, a supervised learning dataset for six-dimensional end pose errors is constructed. Specifically, according to the forward kinematics equation of the UR5 robotic arm, the expected and actual joint angles are converted into Cartesian coordinates to obtain the theoretical and actual end poses. The differences between the two groups of data are calculated to generate six-dimensional pose error labels for model training.

In terms of model design, this paper proposes a physics-aware error modeling framework based on the Transformer encoder, integrating a physics-consistent constraint loss and a nonlinear exponential error amplification strategy. Taking multi-dimensional controller features within the historical time window as input, the model completes linear mapping and position encoding and adopts multi-layer self-attention encoders and regression heads to output the predicted six-dimensional end pose errors at the current moment.

For experimental evaluation, multiple quantitative metrics including MAE and RMSE, as well as compensation improvement rate, are adopted to evaluate the model performance. Meanwhile, typical benchmark models such as MLP, XGBoost and random forest are introduced for comparative experiments. The performance differences in different models in robotic arm error prediction and compensation are fully analyzed to verify the effectiveness and superiority of the proposed method.

Different from existing Transformer-based robotic arm error compensation studies, the core contribution of this paper lies not in modifying the Transformer architecture itself, but in proposing a physics-aware error modeling framework. This framework organically integrates a standard Transformer encoder with the following two dedicated strategies customized for the error characteristics of robotic manipulators: (1) Physical Consistency Constraint Loss Function: Relying on the prior knowledge of manipulator dynamics, we impose a consistency constraint between the direction of predicted error and the direction of joint torque or end-effector velocity during training, so as to guide the model to produce error predictions conforming to physical laws. (2) Nonlinear Exponential Error Amplification Strategy: To tackle the issue of extremely tiny end-effector errors of the manipulator (on the order of 10^−4^ to 10^−5^) and the resulting vanishing gradient of the neural network, a reversible nonlinear exponential transformation is designed to stretch error labels and boost the model’s sensitivity to subtle residual features. The above two strategies are decoupled from the dedicated Transformer architecture and possess favorable model transferability, which can be generalized to other regression models such as MLP and LSTM. This paper quantitatively analyzes the individual contribution of each strategy as well as their synergistic gain via independent ablation experiments.

**Figure 1:**
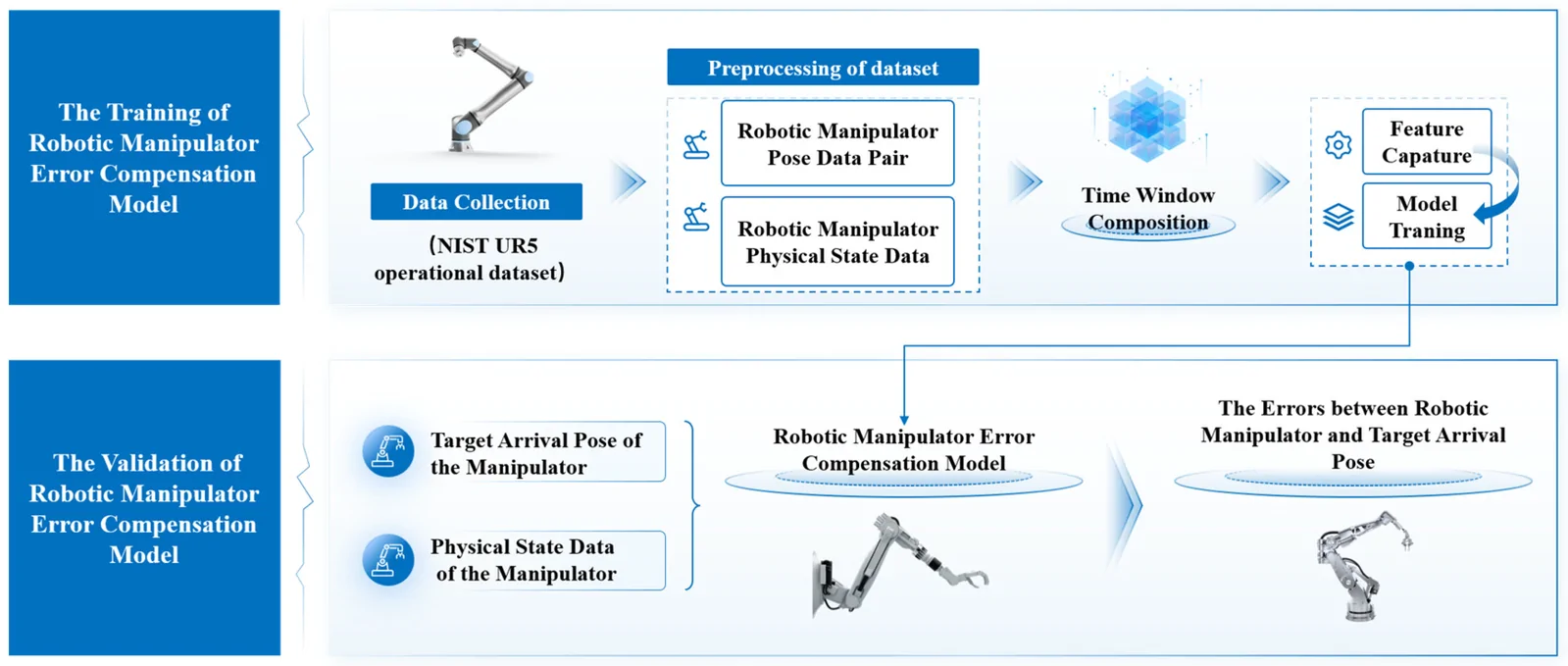
The overall workflow of the proposed method.

Based on the above research background and analysis, this paper selects UR5 industrial robotic manipulator as the study object and proposes a method of robotic end-effector pose error prediction and compensation based on the Transformer. Figure 1 shows the overall working procedures of the proposed method including four stages, which are data collection and preprocessing, temporal sequence feature construction, Transformer model training, error prediction and pose compensation.

This figure shows the complete research procedures from data collection to error compensation. Firstly, the multi-dimensional operating data of UR5 robotic manipulator are collected from the public dataset of NIST (including joint position, velocity, acceleration, current and torque); secondly, we perform preprocessing and feature extraction on the raw data to construct the controller-state feature matrix; and thirdly, we construct historical sequence samples as the Transformer model input by a sliding time window. Then, we train the error prediction model to learn the mapping relationship between historical motion state and end-effector pose errors. Finally, the trained model is applied to the robotic manipulator pose error prediction and performs compensation to the target end-effector pose of the robotic manipulator in the reference stage.

For the convenience of readers, the main symbols in this paper and their definitions are summarized in Table 1.

## 2. Materials and Methods

### 2.1. Data-Driven Error Learning-Based Pose Compensation Algorithm for Manipulators

Different from correction methods based on kinematic models, data-driven compensation algorithms with error learning adopt machine learning models. Instead of relying on explicit physical modeling of error sources, they employ neural networks to learn the mapping relationship between robot states and end-effector pose errors using abundant laboratory measurement data and state information collected during manipulator operation.

The fundamental idea of such methods is as follows: although end-effector errors stem from multiple factors including geometric errors, compliance errors, thermal drift, friction, transmission backlash and control dynamics, these factors leave statistical patterns in data such as joint angles, theoretical poses, velocities, accelerations, torques, currents, payloads and historical motion states. Provided the collected data adequately cover the workspace and working condition distributions, the machine learning model can predict errors, and the predicted errors are fed back as compensation values to target poses or joint commands.

As stated by Chen et al. in their review on data-driven calibration [9], data-driven calibration models reduce the difficulty of modeling complex systems via large-scale datasets, constituting a cost-effective and efficient approach to improve robot accuracy. Their modeling strategies can be categorized into direct spatial distance solving methods, neural network methods, filtering methods and probabilistic methods.

Classified by model type, data-driven compensation approaches can be divided into traditional machine learning, neural networks, deep learning time-series models, probabilistic models and other categories. Spatial interpolation, Kriging, radial basis function (RBF) interpolation and error similarity methods estimate errors at unmeasured target points based on the assumption that errors are approximate at adjacent spatial points or under similar joint configurations. Models such as support vector regression (SVR), Gaussian process regression (GPR), random forests and gradient boosting trees are suitable for tabular controller data and capable of fitting nonlinear feature interactions. Among them [29], Gaussian processes can additionally output prediction uncertainty, yet they suffer high computational overhead when dealing with large-scale datasets.

Back Propagation (BP) neural networks, RBF neural networks, multi-layer perceptrons (MLPs), deep belief networks (DBNs) and other approaches possess powerful nonlinear approximation capability [30]. They can learn the mapping from joint space or pose space to error space without constructing complex explicit error mechanism models. Nguyen et al. proposed a hybrid scheme combining the extended Kalman filter (EKF) with artificial neural networks (ANNs) [31]. In this method, the EKF is first utilized to handle geometric parameter errors, followed by ANNs to predict non-geometric errors [32]. Experiments demonstrated a remarkable improvement in robot positioning accuracy. Such hybrid frameworks embody the merits of integrating model-driven and data-driven methodologies.

In recent years, deep learning has further advanced data-driven error compensation from static mapping to temporal sequence modeling. During continuous operation of industrial manipulators, end-effector errors are determined not only by current joint angles and target poses, but also by velocity, acceleration, friction states, motor currents, control currents, payload variations and historical motion trajectories. If only single-timestep states are fed as model inputs, the model fails to capture time-accumulated error sources including thermal drift, hysteresis, transmission backlash and dynamic control errors.

Sequence models such as recurrent neural networks (RNNs), Long Short-Term Memory (LSTM) [33], Gated Recurrent Unit (GRU) and Transformer leverage historical state information within sliding time windows to predict current errors. Among them, the self-attention mechanism of Transformer adaptively assigns weights across different timesteps and feature dimensions, making it well-suited to capture long-term dependencies and multi-factor coupling relationships in multivariate controller logs [34,35]. The Transformer model adopted later in this paper, together with the comparative baselines including MLP, XGBoost [36] and random forest, are all inspired by the above research paradigm of data-driven error learning.

The core merits of data-driven error learning methods lie in their strong adaptability to complex nonlinear and multi-coupled errors, enabling compensation for residual errors that cannot be fully characterized by traditional kinematic models or single stiffness models. Liu et al. [28] pointed out that intelligent algorithms such as neural networks and deep learning pave the way for full-workspace error prediction; future research can further integrate online measurement equipment with intelligent algorithms to realize online adaptive adjustment of robot poses. Nevertheless, such approaches still suffer from prominent drawbacks as follows: heavy reliance on training samples, high risk of out-of-distribution extrapolation, insufficient physical interpretability, and unstable generalization across different working conditions.

### 2.2. Transformer-Based Error Compensation for Manipulators

#### 2.2.1. Principle of the Transformer Model

**Figure 2:**
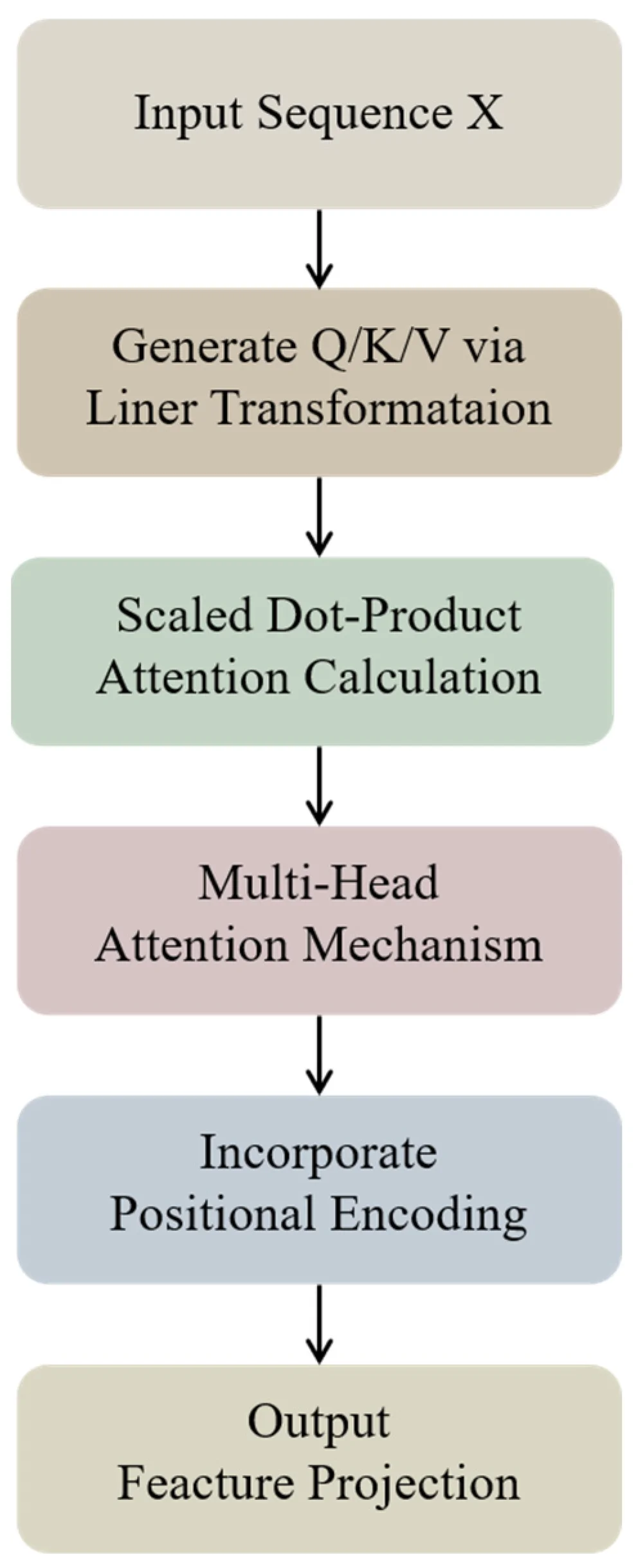
Architecture of the Transformer encoder with multi-head self-attention.

Transformer architecture was originally proposed for sequence modeling tasks in natural language processing. As shown in Figure 2, its core distinction from other neural networks lies in the innovative attention mechanism. Unlike recurrent neural networks (RNNs) that process sequences recursively along the time axis, the Transformer enables parallel computation over the entire input time series. Via learnable attention weights, it learns correlations between any two arbitrary timesteps within the sequence.

In this research, manipulator motion inherently contains temporal features. During continuous operation, motion trajectories and robot states accumulated over a historical period give rise to cumulative errors. This paper holds that such temporally accumulated errors exert non-negligible impacts on subsequent movement. Accordingly, each timestep within the input sequence can reflect the manipulator’s operating states over a preceding time window, and the self-attention mechanism autonomously quantifies how historical input states contribute to end-effector pose errors.

The attention mechanism is formulated as follows. Given the input sequence X, the model first performs linear transformations to obtain the query matrix Q, key matrix K and value matrix V. Scaled dot-product attention is defined as follows:

(1) $$Attention\left(Q,K,V\right)=softmax\left(QK^{T}/\sqrt{d_{k}}\right)V$$

The multi-head attention mechanism projects inputs into multiple subspaces to compute attention outputs in parallel. The outputs of all heads are concatenated and then fed into a linear transformation, which strengthens the model’s ability to represent diverse feature correlations and temporal dependencies. Here, $W^{O}\in R^{\left(h\cdot d_{k}\right)\times d_{model}}$ model denotes the final linear weight matrix of multi-head attention, which conducts dimension reduction and information fusion on concatenated multi-head features. h refers to the number of attention heads, $d_{k}$ is the feature dimension of each single head, and $d_{model}$ represents the hidden feature dimension of the model.

(2) $$MultiHead\left(Q,K,V\right)=Concat\left(head_{1},\ldots ,head_{h}\right)W^{O}$$

The Transformer architecture has no recursive structure adopted by conventional temporal models such as LSTM, so it cannot automatically capture the sequential order of sequences. Additional positional encoding is required to represent the temporal order of input sequences. This paper adopts sine and cosine positional encoding to enable the model to distinguish data at different timestamps within the window.

(3) $$PE_{\left(pos,2i\right)}=sin\left(pos/{10,000}^{2i/d_{model}}\right)$$

(4) $$PE_{\left(pos,2i+1\right)}=cos\left(pos/{10,000}^{2i/d_{model}}\right)$$

$PE\in R^{T\times d_{model}}$ stands for the absolute sine–cosine positional encoding matrix, which injects explicit temporal topological order into the parallel self-attention structure. T is the length of the sliding time window. $pos\in \left[0,T-1\right]$ indicates the absolute time step index of the current sample within the temporal feature window. $i\in \left[0,\left\lfloor d_{model}/2\right\rfloor -1\right]$ represents the dimension index in the spatial feature channel of the positional encoding vector, which differentiates odd and even channels in the feature vector and jointly controls the waveform frequency of sine and cosine mapping functions.

#### 2.2.2. Principle of Manipulator Pose Compensation Based on Transformer

In this paper, the end-effector pose error compensation task of manipulators is formulated as a multi-output time series regression problem. The model takes multi-dimensional operating states of the manipulator over a period of runtime as input and outputs the six-dimensional pose error of the end-effector at the current timestep. The compensation workflow consists of data reading, feature extraction, forward kinematics transformation, label construction, error scaling, time window construction, model training, error prediction, inverse standardization and compensation performance evaluation.

For validation set evaluation, this paper first calculates the raw end-effector pose error of the manipulator without compensation. The trained model is then adopted to predict such errors. Finally, the raw errors are compared with predicted errors to verify whether the model can effectively estimate end-effector pose deviations.

The fundamental principle and core idea of the manipulator pose compensation task lie in utilizing machine learning models to predict potential pose offset errors of the end-effector under the current operating conditions. After obtaining the predicted errors, control commands are adjusted to counteract these deviations, so that the manipulator can accurately reach the target position.

**Figure 3:**
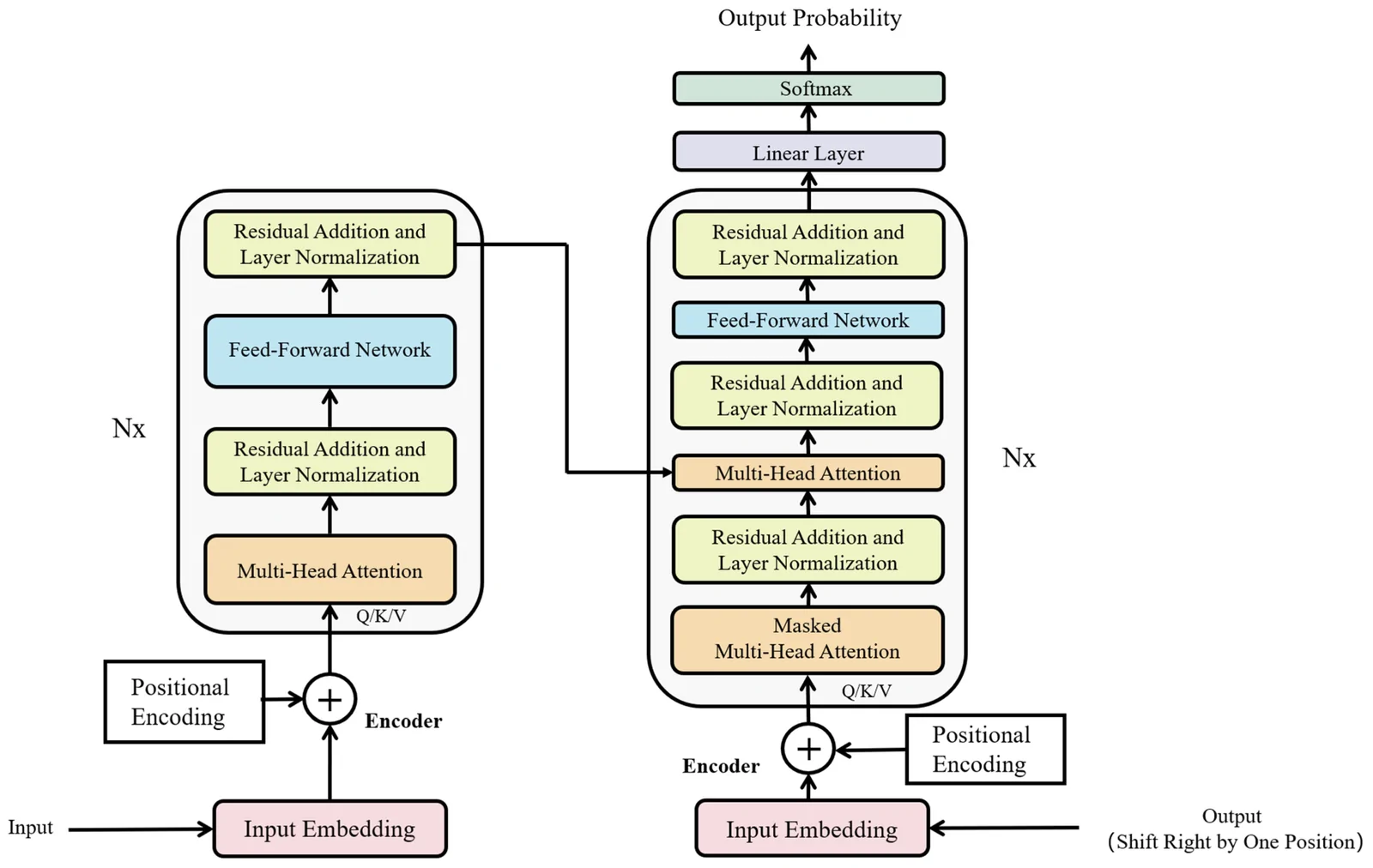
The workflow of pose error prediction and compensation for the robotic manipulator based on the Transformer.

As illustrated in Figure 3 [37], this work adopts multi-dimensional operating state features of the UR5 manipulator as model inputs. Target joint positions together with operating state features such as velocity, acceleration, motor current and joint torque are sequentially assembled into strictly historical time-series feature windows with a fixed length of L. Crucially, each input window contains only controller states from past time steps (from t−L to t−1), excluding all variables at the current time step t. This strict causal structure prevents any potential data leakage that could arise from using control currents or other contemporaneous variables that may correlate with the instantaneous tracking error.

As illustrated in the figure, the model takes a historical time window of length L (spanning multi-dimensional controller features from time step t−L to t−1) as input and outputs the predicted 6-dimensional end-effector pose error at the current time step t. The input window strictly excludes all features at time step t (such as control current, driving torque, etc.) to maintain temporal causality and avoid synchronous data leakage.

Let the input feature vector at timestep t be $x_{t}$; then, a sample can be presented as follows:

(5) $$X_{t}=\left[x_{t-L+1},x_{t-L+2},...,x_{t-1}\right]\in R^{T\times d}$$

where L denotes the length of the time window, and d represents the input feature dimension at each time step. This window exclusively adopts historical controller states prior to time step t and excludes all control information at time step t, so as to guarantee causal prediction and eliminate data leakage caused by synchronous coupling between the control current and the instantaneous tracking error at time t. It is worth emphasizing that the latest time step inside the window is t−1 instead of t. That is to say, when predicting the pose error at time t, the model only relies on historical motion states and control information before time t, without introducing variables such as the control current at time t that have synchronous coupling with the real-time error. This causal lag design guarantees temporal consistency during model training and evaluation and effectively avoids data leakage.

The supervised learning label of the model is the end-effector pose error at the current timestep, which is defined as follows:

(6) $$e_{t}=p_{t}^{\mathrm{act}}-p_{t}^{\mathrm{tar}}={\left[e_{x},e_{y},e_{z},e_{rx},e_{ry},e_{rz}\right]}^{T}$$

The first three components represent translational position errors of the end-effector, while the last three correspond to rotational attitude errors. The mapping relationship to be learned by the Transformer model is equated as follows:

(7) $${\hat{e}}_{t}=f_{\theta }\left(X\right)$$

It should be noted that the error of the model prediction is a pose deviation in Cartesian space. In actual industrial applications, to transfer the compensation value to the robot controller, the corrected end-effector pose pttar+et needs to be converted into joint-space commands via inverse kinematics:

(8) $$q_{t}^{\mathrm{comp}}=IK\left(p_{t}^{\mathrm{tar}}+{\hat{e}}_{t}\right)$$

Here, $IK\left(\cdot \right)$ denotes the inverse kinematics function. The accuracy of the inverse kinematics solution directly determines the precision of compensation commands, and its specific solution method is presented in Section 3.2.6.

That is, using a neural network model with parameter θ, the six-dimensional pose error at the current moment is predicted based on the robotic arm’s motion states over a past period. Unlike conventional fully connected networks, Transformers compute correlations between different time steps through self-attention mechanisms. For the query matrix Q, key matrix K, and value matrix V obtained by mapping the input sequence, the self-attention calculation equation is as follows:

(9) $$Attention\left(Q,K,V\right)=softmax\left(QK^{T}/\sqrt{d_{k}}\right)V$$

The application of attention mechanisms enables the model to focus on historical state information and input features that significantly influence current errors, thereby better capturing error sources during robotic arm motion, including temporal dependencies and coupling effects among multiple features. To preserve temporal information in the model input, this paper introduces positional encoding after feeding in the current features, using sine and cosine variations so that the model can distinguish the order of different time steps. After deep feature extraction through multiple Transformer encoder layers, the vector at the final time step of the output sequence is extracted as the feature mapping. This vector, having undergone attention aggregation across the entire sequence, effectively represents the global motion characteristics within the current time window and serves as input to the regression layer for six-dimensional error prediction.

#### 2.2.3. Model Training

This paper adopts the Transformer model as the core error prediction model. Its architecture is illustrated in Table 2, consisting of an input projection layer, a positional encoding layer, stacked multi-layer Transformer encoders and a regression output head.

**Figure 4:**
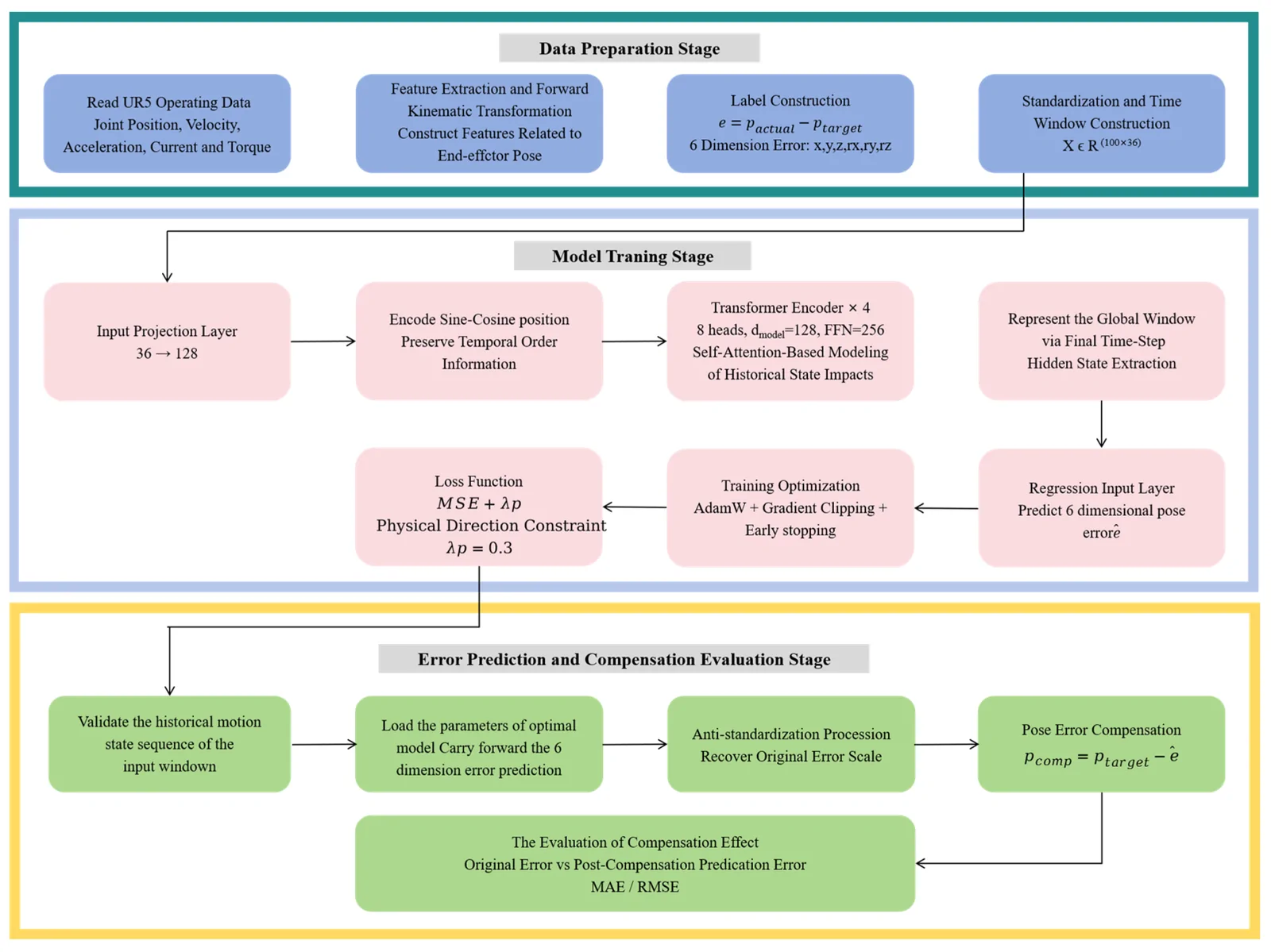
Schematic diagram of the position compensation process for the mechanical arm.

As illustrated in Figure 4, the input layer linearly maps the 36-dimensional raw features of each timestep into a 128-dimensional hidden space. The positional encoding layer injects sequential order information into the feature sequence. The encoder stack contains 4 layers of Transformer encoder; each layer is equipped with 8 attention heads, a feed-forward network, residual connections, layer normalization and Dropout. Finally, the hidden state corresponding to the last timestep of the sequence is extracted to represent the entire time window, which is fed into the regression head to output the predicted six-dimensional pose error.

Each input sample is a tensor of size L×d=100×36, which strictly employs features from historical time steps t−L to t−1 to predict the error at time t, excluding any controller features at time t (including control current, driving torque, etc.) to prevent data leakage caused by synchronous coupling.

Unlike MLP, which flattens the time window directly, the Transformer explicitly models the relationships between different timesteps along the sequence dimension. For the manipulator error prediction task, the end-effector error at the current timestep may be correlated with variations in velocity, acceleration and driving current over the preceding period. The self-attention mechanism of the Transformer can automatically focus on more critical historical states according to the demands of error prediction.

This paper mainly adopts the mean squared error (MSE) loss function as the objective of supervised learning. Let the predicted error of the model be $\hat{y_{i}}$ and the ground-truth error label be $y_{i}$, with the total number of samples denoted as n, the MSE loss function is equated as follows:

(10) $$MSE=\frac{1}{n}\sum_{i=1}^{n}{\left(\hat{y_{i}}-y_{i}\right)}^{2}$$

The optimizer used is AdamW (2017 version) [38,39], with a learning rate of $2\times 10^{-4}$, weight decay of $1\times 10^{-4}$, a training batch size of 256, and a maximum of 100 training epochs. To enhance training stability, gradient clipping is applied during backpropagation; to prevent overfitting, an early stopping mechanism is employed—training is halted when the validation loss fails to decrease significantly for 20 consecutive epochs, and the model parameters achieving the best performance on the validation set are saved.

For the robotic manipulator error prediction task, the purely data-driven MSE loss function only concentrates on the numerical precision of prediction results, while it cannot ensure the physical validity of predicted errors. For example, the model may produce predicted errors whose magnitude is close to ground-truth error, yet their directions are completely contradictory to the directions of current joint torque and end-effector velocity. Such physically inconsistent predictions will trigger conflicts between controller outputs and the dynamic properties of the manipulator during actual compensation, which further deteriorates the stability and safety of the compensation process. To solve this problem, this paper introduces a physical consistency constraint based on the following physical prior: the direction of manipulator end-effector error has an inherent physical correlation with its motion states (joint torque, end-effector velocity). When the joint driving torque is large, the error induced by joint flexible deformation develops along the acting direction of torque; when the end effector moves at a high speed, the error brought by inertial effects follows the velocity direction. Guided by this physical prior, a physics-aware regularization term is constructed in this work, which constrains the directional consistency between the predicted error and joint torque/end-effector velocity via cosine similarity.

In this experiment, in addition to the standard mean squared error (MSE) loss, a physics-aware regularization term is introduced to improve the reliability of predictions in real robotic arm control scenarios. This loss function imposes soft constraints on the relationship between prediction errors and the directions of joint torques and end-effector velocities, based on the standard MSE. Specifically, for each training sample, the MSE between the predicted error $\hat{y_{i}}$ and the true error $y_{i}$ remains unchanged, while the alignment between the prediction error direction and the directions of the joint torque vector and end-effector velocity vector is computed using cosine similarity, forming a physical penalty term. The final total loss function L is a weighted combination of the MSE and the physical term:

(11) $$L=\frac{1}{n}\sum_{i=1}^{n}{\left(\hat{y_{i}}-y_{i}\right)}^{2}+\frac{\lambda_{p}}{n}\sum_{i=1}^{n}\left(1-\cos\theta_{i}\right)$$

Here, $\theta_{i}$ represents the angle between the direction of the prediction error and the corresponding joint torque and velocity directions, and $\lambda_{p}$ is a weighting coefficient balancing the MSE and the physical constraint penalty, set to 0.3 in this experiment. This design aims to guide the predicted errors output by the model to align with the actual dynamic characteristics of the manipulator. It therefore improves the accuracy and stability of compensation, while avoiding overly strict restrictions on the training process to preserve strong generalization performance of the model. Experimental results show that after introducing physical constraints, the MAE and RMSE of the Transformer for end-effector pose error prediction both decline to a certain extent. The performance improvement is especially prominent under heavy-load and high-speed motion working conditions. In this paper, the weight of the physical constraint is set to $\lambda_{p}=0.3$. This value is determined via grid search on the validation set over the search range $\left[0.0,0.1,0.2,0.3,0.4,0.5\right]$. The validation-set MAE decreases continuously when $\lambda_{p}$ increases from 0 to 0.3. Once $\lambda_{p}>0.3$, the physical constraint dominates the overall loss function excessively, which inversely causes the MAE to rise. Consequently, $\lambda_{p}=0.3$ achieves the optimal balance between prediction accuracy and physical consistency.

All experiments in this work are conducted in Google Colab (2026.01 version). The experimental codes are programmed in Python with Python 3.11 as the runtime environment, and an NVIDIA A100 GPU is utilized for model training.

## 3. The Introduction and Possession of Data Assemble

### 3.1. Dataset Construction

#### 3.1.1. Kinematic Model and D-H Parameters of the UR5 Manipulator

To establish an accurate mapping between the joint space and the Cartesian space of the UR5 manipulator, this section presents its kinematic model and the corresponding D-H (Denavit–Hartenberg) parameters.

All values in this table are taken from the official UR5 parameters provided by Universal Robots [40].

The UR5 robotic manipulator is constructed of six revolute joints connected in series (from $J_{1}$ to $J_{6}$); every joint contributes one rotational degree of freedom around its joint axis. To describe the relative pose relationship between adjacent links, this paper adopts the Modified DH Convention [41] to establish the forward kinematic model of the UR5. Under this convention, the homogeneous transformation matrix between coordinate frames of adjacent link i−1 and *i* is uniquely determined by four D-H parameters ($\alpha_{i-1},a_{i-1},d_{i},\;and\;\theta_{i}$):

(12) $$T_{i}^{i-1}=\mathrm{Rot}\left(x_{i-1},\alpha_{i-1}\right)\cdot \mathrm{Trans}\left(x_{i-1},a_{i-1}\right)\cdot \mathrm{Rot}\left(z_{i},\theta_{i}\right)\cdot \mathrm{Trans}\left(z_{i},d_{i}\right)$$

Expressed in matrix form:

(13) $$T_{i}^{i-1}=\left[\begin{array}{cccc} \cos\theta_{i} & -\sin\theta_{i} & 0 & a_{i-1}\\ \sin\theta_{i}\cos\alpha_{i-1} & \cos\theta_{i}\cos\alpha_{i-1} & -\sin\alpha_{i-1} & -d_{i}\sin\alpha_{i-1}\\ \sin\theta_{i}\sin\alpha_{i-1} & \cos\theta_{i}\sin\alpha_{i-1} & \cos\alpha_{i-1} & d_{i}\cos\alpha_{i-1}\\ 0 & 0 & 0 & 1 \end{array}\right]$$

Based on the geometric parameters of the UR5 officially published by Universal Robots [42], the modified D-H parameters adopted by this paper are listed in Table 3. Among them, $\alpha_{i-1}$ denotes the link rotational angle, $a_{i-1}$ denotes the link length, $d_{i}$ denotes the link offset, and $\theta_{i}$ denotes the joint angle. The six joint angles from $\theta_{1}$ to $\theta_{6}$ are the control input variables of UR5. The motion range of each joint is listed in Table 3. Only the elbow joint ($J_{3}$) is limited to ±160° whereas all other joints can rotate by ±360°.

Based on the above D-H parameters, the forward kinematics of the UR5 manipulator can be expressed as the product of the transformation matrices of each link:

(14) $$T_{6}^{0}\left(\theta \right)=T_{1}^{0}\left(\theta_{1}\right)\cdot T_{2}^{1}\left(\theta_{2}\right)\cdot T_{3}^{2}\left(\theta_{3}\right)\cdot T_{4}^{3}\left(\theta_{4}\right)\cdot T_{5}^{4}\left(\theta_{5}\right)\cdot T_{6}^{5}\left(\theta_{6}\right)$$

Here, $T_{6}^{0}\in SE\left(3\right)$ denotes the homogeneous transformation matrix of the end-effector with respect to the base coordinate frame. Its translational component corresponds to end-effector position coordinates $\left(x,\;y,\;and\;z\right)$, while the rotational component corresponds to end-effector pose. This forward kinematics model serves as the theoretical foundation for calculating the end-effector pose error labels in Section 3.2.2 of this paper (Equations (16) and (17)).

#### 3.1.2. Experimental Devices and Sensor Configuration

**Figure 5:**
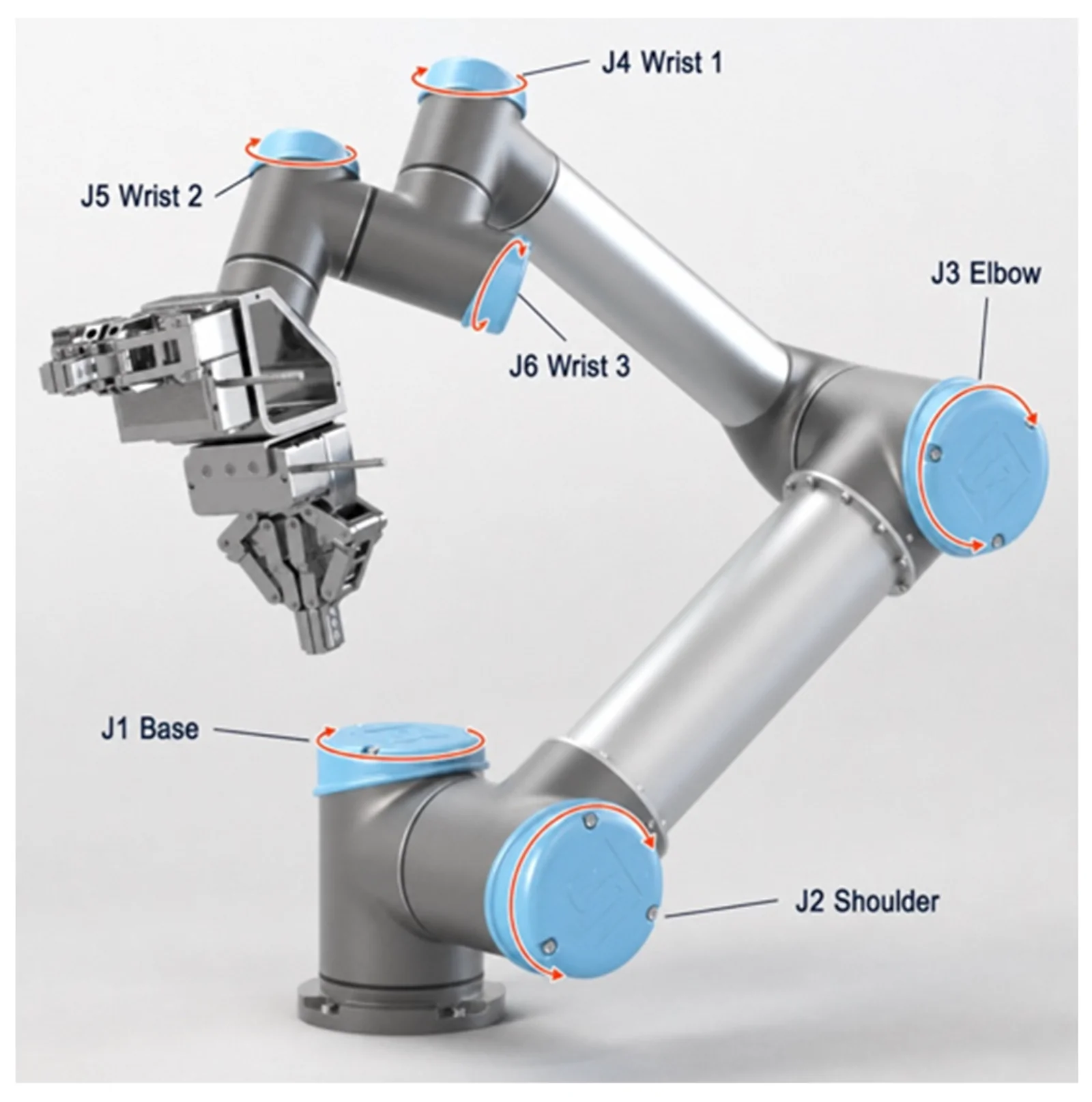
Schematic diagram of the six-degree-of-freedom UR5 robotic manipulator.

As shown in Figure 5, the UR5 robotic manipulator is a 6-degree-of-freedom (6-DOF) serial collaborative robot developed by Universal Robots. All six joints are revolute joints, denoted as J_1_ to J_6_ from the base to the end-effector. Specifically, J_1_ is the base joint (waist rotation), J_2_ is the shoulder joint, J_3_ is the elbow joint, and J_4_, J_5_, and J_6_ form the three wrist joints. Featuring a compact structure, flexible motion, easy deployment, and high safety, it is widely adopted in industrial scenarios including manufacturing, assembly, packaging, logistics handling, and precision manipulation. Each joint provides one rotational degree of freedom, which gives the UR5 a total of six degrees of freedom. Connected by links, the joints form a serial kinematic chain, enabling position control and limited orientation control of the end-effector in three-dimensional space. Each joint has a motion range of ±360°, except for the elbow joint (J_3_), which is limited to ±160°. Benefiting from excellent repeatability and widespread industrial adoption, the UR5 is frequently used as a standard experimental platform for research on robotic kinematics analysis, dynamic modeling, trajectory optimization, and error compensation.

UR5 is a 6-DOF serial collaborative robot developed by Universal Robots. Its six revolute joints are denoted as J_1_ (base joint), J_2_ (shoulder joint), J_3_ (elbow joint), J_4_ (wrist joint 1), J_5_ (wrist joint 2), and J_6_ (wrist joint 3), from the base to the end-effector. Each revolute joint contributes one rotational degree of freedom, giving a total of six degrees of freedom. The motion range of each joint is ±360°, except for the elbow joint (J_3_), which is limited to ±160°.

From the perspective of kinematics, the end position and orientation of the UR5 robotic arm are jointly determined by six joint angles. The forward kinematics process calculates the position and orientation of the end effector in the base coordinate system based on the joint angles; the inverse kinematics process solves the corresponding joint angles based on the desired end position. Relevant studies have pointed out that in the real-time motion analysis of the UR5 robotic arm, the joint angles, joint velocities, and the position of the tool center point in the x, y, and z directions are commonly used as important variables to describe the motion state of the robot. These data can reflect the motion laws and dynamic characteristics of the robotic arm in different time periods. From the perspective of dynamics, the UR5 robotic arm is affected not only by joint angles and velocities but also by acceleration, torque, inertia, gravity, and external loads during its movement. Conducting real-time kinematics and dynamics analysis of the UR5 robotic arm helps evaluate the motion efficiency, control accuracy, and task execution stability of the robotic arm in actual operation; changes in joint angles can reflect the spatial configuration changes in the robotic arm, fluctuations in joint velocities can reflect the dynamic adjustment of the robotic arm’s motion state, and the trajectory of the end tool point directly reflects the spatial motion accuracy during the execution of the task. Therefore, the UR5 robotic arm is not only suitable for studying basic forward and inverse kinematics problems but also for studying the relationship between multi-source operation data and end position error. This paper takes the UR5 robotic arm as the research object, mainly because its structure is typical, data recording is complete, and the public data set contains multi-dimensional operation information such as joint positions, velocities, current, torque, temperature, and end position. The detailed experimental setup, sensor configuration, and data acquisition procedure of the NIST dataset are described in the following subsection. These data can comprehensively reflect the motion state of the robotic arm under different working conditions, providing a foundation for constructing a data-driven end position error prediction and compensation model. In the subsequent part of this paper, based on the operation data of the UR5 robotic arm, the end position error labels will be constructed using the forward kinematics method, and the end six-dimensional position error of the robotic arm will be predicted and compensated by combining the Transformer model.

**Figure 6:**
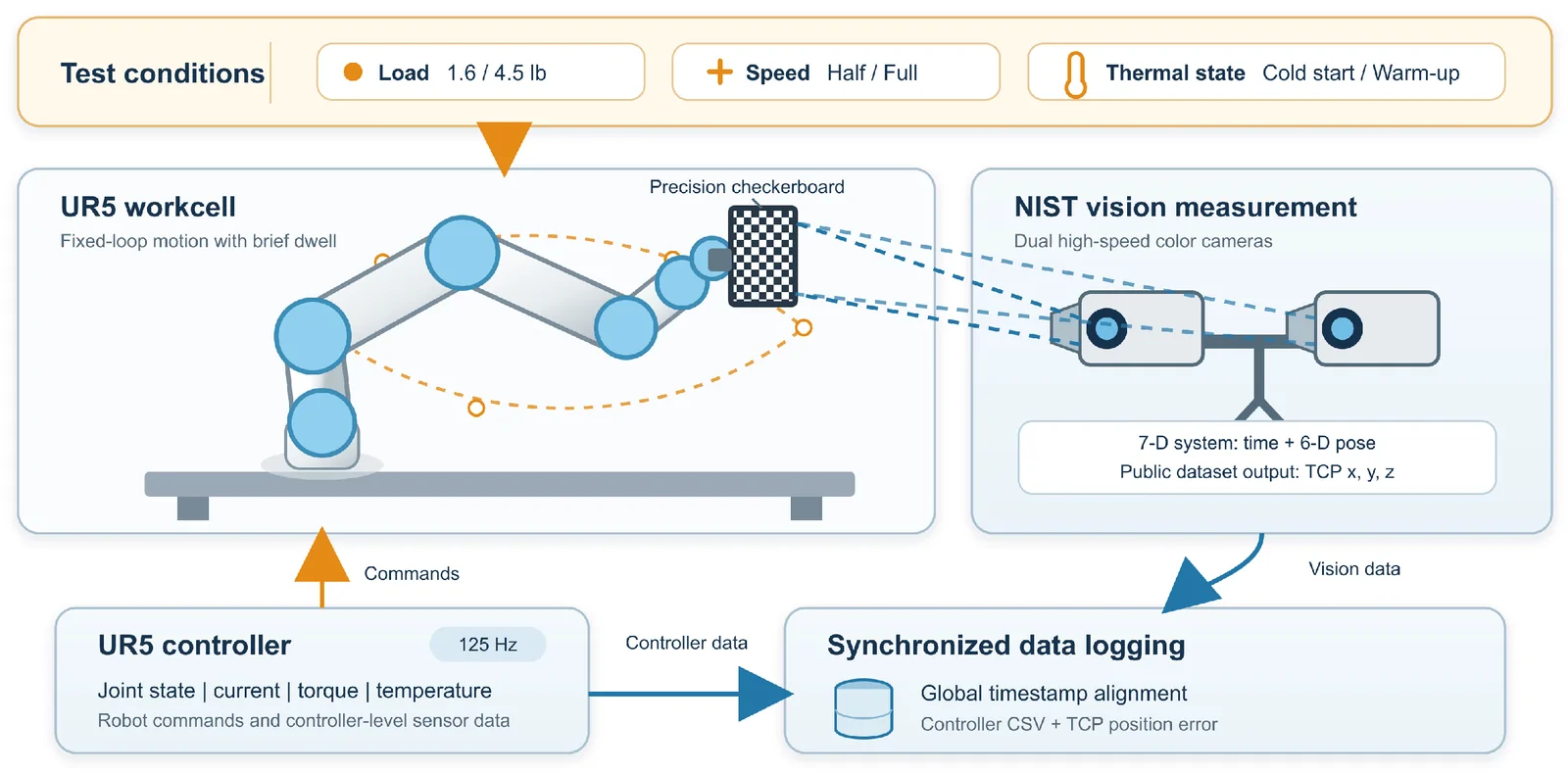
Schematic diagram of the NIST robotic manipulator experimental device for pose accuracy degradation measurement.

As shown in Figure 6, all data adopted by this research are from the dataset Robotic Manipulator Pose Accuracy Degradation Measurement publicly published by Engineering Laboratory Intelligent System Department, American National Institute of Standards and Technology (NIST) [40]. This dataset is constructed based on experiments conducted with Universal Robots UR5 robotic manipulator, aiming to support the health evaluation and pose precision degradation analysis of the robot system by collecting operational data under diverse working conditions.

The core experimental device is a UR5 6-DOF serial collaborative manipulator. Its controller-level sensory data—including target/actual joint positions, velocities, accelerations, torques, currents, control currents, and temperatures—are directly acquired from the UR5 controller output, with a sampling frequency of 125 Hz. The controller-level data can contribute to the root cause analysis of the performance degradation by providing the underlying clues, thereby unveiling the impact of factors including temperature loading and velocity on pose accuracy.

For end-effector pose measurement, NIST independently developed a 7-degree-of-freedom (7-DOF) measurement system to acquire the actual pose of the robotic manipulator’s Tool Central Point (TCP). This system can acquire the position of TCP (x,y,z) in three-dimensional space with a high precision, and the end-effector position deviation is calculated by comparison with the theoretical position solved using nominal kinematic parameters. In addition, NIST has developed a vision-based 6-DOF sensing system that measures the full six-dimensional pose, including x, y, z, yaw, pitch, and roll. The information is used for robot’s precision degradation evaluation and improvement. This visual sensing system has been tested on the UR5 robotic manipulator, which can monitor and evaluate the precision degradation of robots under various conditions of load, velocity and temperature.

In the process of data collecting, the UR5 robotic manipulator operated along the predefined trajectory, approached and stopped at randomly selected waypoints within the task space. Experiments were conducted under various conditions, including temperature (approximately 25 °C at cold start, rising to about 35 °C after two hours of operation), end-effector payload (1.6 lb and 4.5 lb), and motion velocity (half and full speed). Each experiment was repeated multiple times to ensure statistical significance and reproducibility of the data. The detailed information of the controlled variables was recorded in the header file (UR5TestResult_header.xlsx) attached to the dataset.

The above-mentioned experimental devices and sensor configuration ensure the dataset can comprehensively reflect the error patterns of the robotic manipulator under various environmental and operational conditions, providing abundant and reliable feature information for the subsequent data-driven end-effector pose error prediction and compensation model.

#### 3.1.3. Dataset Description

The experimental data in this paper comes from the “Degradation Measurement of Robot Arm Position Accuracy” dataset publicly available in the Engineering Laboratory of Intelligent Systems of the National Institute of Standards and Technology (NIST) [40]. As shown in Table 4, based on this, using the Universal Robots UR5 robotic arm as the experimental object, the health evaluation of the robot system and the decline in position accuracy are analyzed. A set of data were collected using a self-developed method. The dataset contains operation data under different loads and speeds, with a sampling frequency of 125 Hz, which can well reflect the detailed information of the robotic arm’s movement.

Table 4 provides a reference for the meaning of the dataset. Each CSV file in the dataset contains multiple variables recorded by the robot controller, including robot time, target and actual joint positions, target joint velocities, accelerations, torques, currents, joint control currents, temperatures, tool center point position, TCP force, etc. This paper mainly conducts modeling experiments using typical working condition data such as 1.6 lb and 4.5 lb loads, half-velocity and full-velocity and focuses on analyzing the relationship between multi-dimensional controller characteristics and end position error.

### 3.2. Analysis and Preprocessing of Dataset Characteristics

#### 3.2.1. Analysis of Dataset Characteristics

During the operation of the robotic arm, the end-point error is not only related to the current target joint position, but also affected by dynamic variables such as speed, acceleration, torque, and current. When constructing the features, this paper retains multiple types of variables that can reflect the movement and driving state of the robotic arm. The main input features include the position, velocity, acceleration, torque, target joint current, joint control current of the target joint, and data for each type of variable are available for the six joint channels from J1 to J6. The feature dimension for a single time step input is 36 dimensions.

When collecting this dataset, certain variable control was implemented. The UR5 robotic arm dataset used in this paper fully considered the influence of various control variables on the end-point pose error during the collection process to ensure the reliability and analyzability of the data. In the experiments, factors such as temperature, end-point load, running speed, and the number of repeated experiments were managed as control factors. Temperature changes affect motor thermal drift, gear friction, and controller response, thereby influencing the end-point pose; different end-point loads cause joint flexibility and link elastic deformation, resulting in deviations in the end-point position and posture; different motion speeds change the joint dynamic response and inertial effect, further affecting the end-point error [43]. To ensure the statistical significance and repeatability of the experimental data, multiple repeated collections were conducted for each condition, and the joint angle, velocity, current, torque, temperature, and end-point pose were recorded at a high frequency (125 Hz). Through systematic management of these control variables, the dataset can reflect the error patterns of the robotic arm under different environments and operating conditions, providing rich and reliable feature information for subsequent data-driven end-point pose error prediction and compensation models and supporting the generalization ability of the model in different conditions.

#### 3.2.2. Dataset Preprocessing

To utilize the ability of the Transformer model to process sequence data, this paper generates the training set using a sliding time window [44]. For a continuous input window of length L, the model input is a multi-variable state sequence over L time periods, and the supervisory label is the six-dimensional end-point pose error of the last time period of the window. In main experiments, the window length is set to L=100, and the model predicts the error at the current moment based on the robotic arm’s running state in the previous period.

(15) $$X_{t}=\left[x_{\left\{t-L+1\right\}},x_{\left\{t-L+2\right\}},\ldots ,x_{t}\right],y_{t}=e_{t}$$

This sliding window construction method guarantees a strict causal temporal relationship between input features and prediction labels by extracting all features from historical time steps before time t, while excluding variables synchronously coupled with $e_{t}$ (e.g., the control current at time t), thereby fundamentally preventing data leakage. The original controller data of NIST contain target joint angles and actual joint angles. Since this paper focuses on the pose error of the end effector [45], it is necessary to map the joint space data to the Cartesian space. In this study, the forward kinematics calculations of target joint angles and actual joint angles are conducted separately, and the difference between the target end pose $p_{t}^{\left\{tar\right\}}$ and the actual end pose $p_{t}^{\left\{act\right\}}$, denoted as $e_{t}$, is obtained as the supervision learning label.

(16) $$p_{t}^{\left\{tar\right\}}=FK\left(q_{t}^{\left\{tar\right\}}\right),p_{t}^{\left\{act\right\}}=FK\left(q_{t}^{\left\{act\right\}}\right)$$

(17) $$e_{t}=p_{t}^{\left\{act\right\}}-p_{t}^{\left\{tar\right\}}=\left[e_{x},e_{y},e_{z},e_{\left\{rx\right\}},e_{\left\{ry\right\}},e_{\left\{rz\right\}}\right]$$

Here, the position error is represented by the three components x, y, and z, while the attitude error is approximately represented by the difference in the rotation vectors rx, ry, and rz. This representation method is consistent with the common mechanical arm controllers and the commonly used tool position and orientation expression methods, which is convenient for the subsequent implementation of the intelligent compensation system.

#### 3.2.3. Data Standardization and Design of the Test Set

The data of the robotic arm controller encompass various different physical quantities, and the scales and numerical ranges of different features vary significantly. For instance, the joint angle is typically expressed in radians, while joint velocity, joint acceleration, current, torque, and temperature have distinct physical units and ranges of variation. If these raw features are directly input into the neural network, the features with larger numerical ranges will occupy a greater weight during matrix operations and gradient propagation, while features with smaller numerical ranges but related to errors may be weakened, thereby affecting the model’s comprehensive utilization of multiple source features.

From the perspective of neural network optimization, the goal of model training is to continuously update the parameters through backpropagation, so as to gradually reduce the loss function. Let the input feature be $x_{j}$ and the model parameter be $w_{j}$. In a simple linear mapping, there is the following:

(18) $$\hat{y}=\sum_{j=1}^{m}w_{j}x_{j}+b$$

Here, $\hat{y}$ represents the predicted output of the model, m denotes the dimension of the input features, and b is the bias term. When the numerical scales of different features $x_{j}$ vary significantly, the corresponding parameter gradients will also be affected by the input scales. For the mean squared error loss function ℒ:

(19) $$L=\frac{1}{n}\sum_{i=1}^{n}{\left({\hat{y}}_{i}-y_{i}\right)}^{2}$$

Here, n denotes the number of samples, ${\hat{y}}_{i}$ is the predicted value of the *i*-th sample, and $y_{i}$ is the corresponding true value. $x_{ij}$ represents the *j*-th feature of the *i*-th sample. The gradient of $w_{j}$ can then be expressed as follows:

(20) $$\frac{\partial L}{\partial w_{j}}\;=\frac{2}{n}\sum_{i=1}^{n}\left({\hat{y}}_{i}-y_{i}\right)x_{ij}$$

From the above equation, it can be seen that the magnitude of the gradient is directly related to the numerical scale of the input feature $x_{ij}$. When the numerical range of certain features is much larger than that of other features, the corresponding gradient may also be larger, thereby dominating the parameter update process. This will cause the model training process to be more sensitive to large-scale features and make it difficult to fairly learn the relationship between different physical quantities and the terminal error. Therefore, in the multi-source controller data modeling task, it is necessary to standardize the input features.

The mechanical arm controller data contain multiple physical quantities, and the differences in dimension and numerical range among different features are significant. For example, the value ranges of joint angles, velocities, currents, torques, and temperatures are different. If directly input into the neural network, it may lead to unstable training processes or certain features dominating the gradient update. Therefore, this paper uses StandardScaler to standardize the input features and labels.

(21) $$x^{\prime }=\frac{\left(x-\mu_{train}\right)}{\sigma_{train}}$$

To ensure the authenticity of the experimental evaluation, the standardized parameters were only fitted on the training set and then applied to the validation set and test set, avoiding the leakage of test set distribution information into the model training process. The dataset division was carried out using the method of chronological division. The proportions of the training set, validation set and test set were 70%, 15% and 15% respectively. The data loaders were all set to shuffle=False. This setting can more realistically simulate the application scenario where the model uses historical data to predict future moment errors.

Here, $\mu_{train}$ and $\sigma_{train}$ represent the mean and standard deviation of the features in the training set. After standardization, different features are transformed into distributions with approximately zero mean and unit variance, making data with different units such as joint angles, velocities, currents, and torques more similar in the numerical scale. This not only helps improve the stability of model training but also accelerates the convergence speed of the optimizer, enabling the model to more fully learn the influence of different input features on the end position error.

At the same time, this paper also standardizes the error labels. Since the end position error of the robotic arm has relatively small values, if the original error is directly used as the supervision label, the loss function value and gradient may be small, which is not conducive to model training. After standardizing the labels, the output target can be placed in a more suitable numerical range for neural network optimization, improving the model’s ability to learn small error changes. After the model prediction is completed, the corresponding mean and standard deviation are used to reverse-standardize the prediction results and restore them to the real physical scale for evaluation of the compensation effect.

It should be noted that to ensure the authenticity of the experimental evaluation, the standardization parameters can only be calculated from the training set and cannot be used from the validation set or test set for fitting.

As shown in Table 5, it can be observed that without standardization, the model will be completely unable to learn the intrinsic characteristics of the data to predict the errors. Therefore, standardization is an indispensable step in this experiment. Due to the different physical dimensions of different labels and the large gap in numerical scales, if standardization is not performed, the model will have difficulty learning and fitting the errors.

For the manipulator pose error prediction task, the end-effector pose error is generally at the millimeter or sub-millimeter level (on the order of 10^−4^ to 10^−5^), whose magnitude is far smaller than that of input features (joint angles range from −π to π, and current is at the ampere level). When the raw error is directly adopted as the supervision label, the loss function yields an extremely small value (on the order of $10^{-8}$ to $10^{-10}$ for MSE), accompanied by correspondingly weak gradients during backpropagation. In deep Transformer architectures, such feeble gradients are prone to vanishing gradient after multi-layer backpropagation, making the model hard to learn error features effectively and resulting in insufficient sensitivity to small-magnitude errors. To tackle this issue, inspired by the dynamic range compression/expansion concept in signal processing, we designed a reversible nonlinear exponential error amplification strategy. This strategy amplifies the error magnitude while preserving the original error ordering, generating more distinct gradients for error signals in the feature space and boosting the network’s ability to capture subtle error features. Meanwhile, the transformation strictly satisfies monotonicity and reversibility, so that the model prediction can be restored to the original physical scale in a lossless manner.

During the movement of the robotic arm, the actual position of the end effector usually deviates from the ideal situation at a very small scale. Because this error is too small, it will lead to a weak error gradient in neural network training, making it difficult for the model to effectively capture these small nonlinear errors and dynamic features. At the same time, it is also prone to causing the error to be drowned in the noise of feature extraction and calculation. To solve this problem, this paper proposes a nonlinear error amplification method based on exponential mapping. The main mathematical basis of the method is the reversible exponential function change, which amplifies the small differences between the actual position and the ideal position, thereby enhancing the model’s sensitivity to errors and improving the convergence and prediction accuracy of the network.

To amplify the error, a control parameter α (where α>0) for the transformation intensity is defined, and the original error is subjected to a nonlinear exponential transformation through the following equation to obtain the amplified error e′:

(22) $$e^{\prime }=sgn\left(e\right)\cdot \left(e^{\alpha \left|e\right|}-1\right)$$

Through the above transformation, the minor error fluctuations in the original data have been significantly amplified, enabling the neural network to more easily learn the relationship between the error and the input features. At the same time, in order to accurately evaluate the true prediction accuracy of the model, the transformation must strictly satisfy the reversibility of the mathematical transformation. Since the exponential function transformation is strictly monotonically increasing within its domain, the above mapping is reversible. After obtaining the model’s predicted output value $e_{pred}^{\prime }$, the inverse function of the transformation is used, and logarithmic operations are performed to restore the error to the original scale, obtaining the original physical prediction result $e_{pred}$:

(23) $$e_{pred}=sgn\left(e_{pred}^{\prime }\right)\cdot \frac{\ln\left(\left|e_{pred}^{\prime }\right|+1\right)}{\alpha }$$

In this paper, the exponential amplification coefficient α=5.0 is determined via grid search on the validation set over the search range $\left[1.0,2.0,3.0,5.0,8.0,10.0\right]$. Insufficient amplification is observed when α is too small (<3.0); conversely, an overly aggressive transformation occurs if α is too large (>8.0), which increases the reconstruction error. The validation set achieves the minimum MAE at α=5.0, hence this value is selected as the optimal hyperparameter. This paper has established data processing flow from residual extraction, exponential stretching, and logarithmic mapping restoration. This method enhances the significance of minor errors in the feature space through nonlinear exponential transformation, effectively solving the problem that neural networks are insensitive to small features and difficult to converge. At the same time, this transformation mathematically satisfies reversibility, and the use of logarithmic reversible transformation ensures the rigor of the experiment.

#### 3.2.4. Data Correlation Analysis

The dataset of this study includes various features. Different features have different degrees of influence on the final error. Therefore, in order to verify the rationality of the selected input features, this paper visually analyzed the Pearson correlation coefficients between joint current, joint speed and the six-dimensional end position error. The x, y, z, rx, ry, rz six error components are on the horizontal axis of the heatmap, and the joint current or speed feature is on the vertical axis. The value in each cell represents the degree of linear correlation between the corresponding feature and the error component.

**Figure 7:**
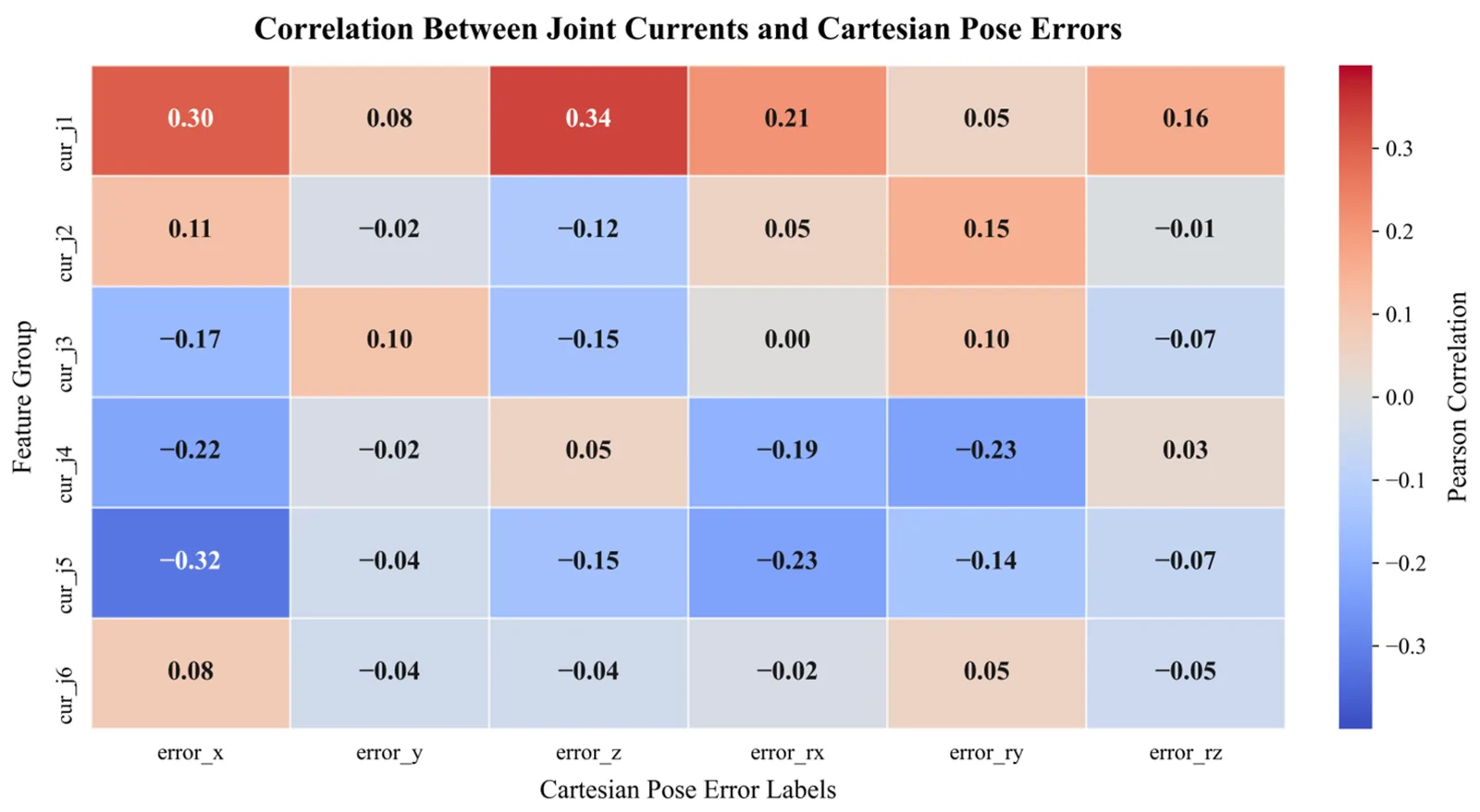
Heat map showing the correlation between joint current and end position error (halfspeed;playload4.5bl).

**Figure 8:**
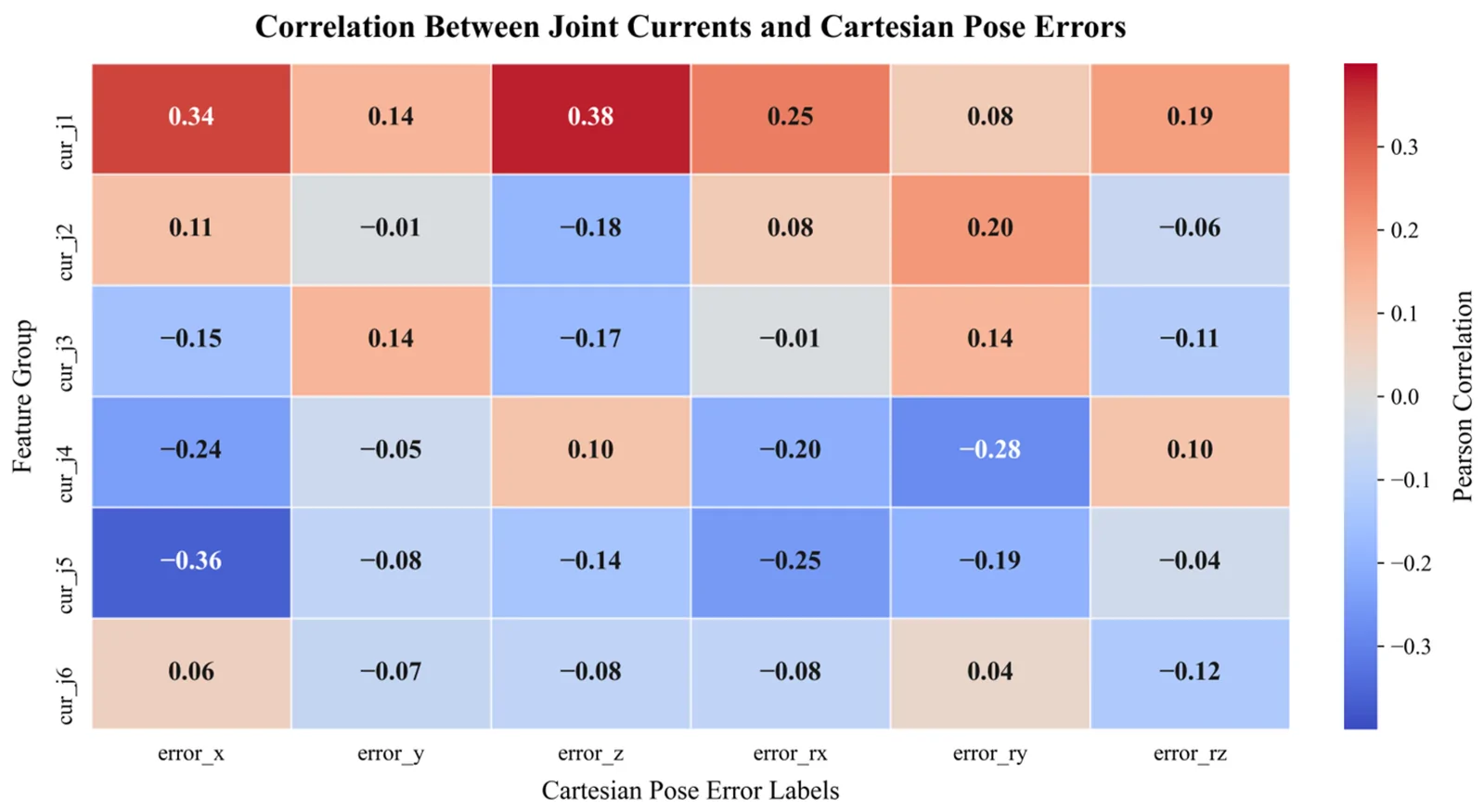
Heat map showing the correlation between joint current and end position error (halfspeed;playload1.6bl).

As shown in Figure 7 and Figure 8, joint currents have a certain correlation with end-effector pose errors, yet currents from different joints exert distinct impacts on each error component. Under two different load working conditions, the currents of partial joints present significant correlations with end-effector position and pose errors. For instance, the current of Joint J1 has a strong positive correlation with ${error}_{x}$ and ${error}_{z}$, indicating that variations in the driving current of the base joint can remarkably affect the spatial position errors of the end effector. By contrast, the current of Joint J5 shows a moderate negative correlation with ${error}_{x}$ and ${error}_{rx}$, which reveals that fluctuations in the operating state of the wrist joint also interfere with the end-effector’s pose and positioning accuracy.

Since joint current can reflect motor output torque, load variations and the operating state of the drive system to a certain extent, its correlation with end-effector pose errors proves that robotic arm errors stem not only from geometric parameter deviations but are also collectively affected by driving loads, joint forces and the dynamic characteristics of actuators. Meanwhile, the magnitudes of correlation coefficients shift under different load conditions, which demonstrates that load variation alters the coupling relationship between joint currents and end-effector errors. This provides a solid experimental basis for the subsequent research that takes joint currents as input features of the model.

**Figure 9:**
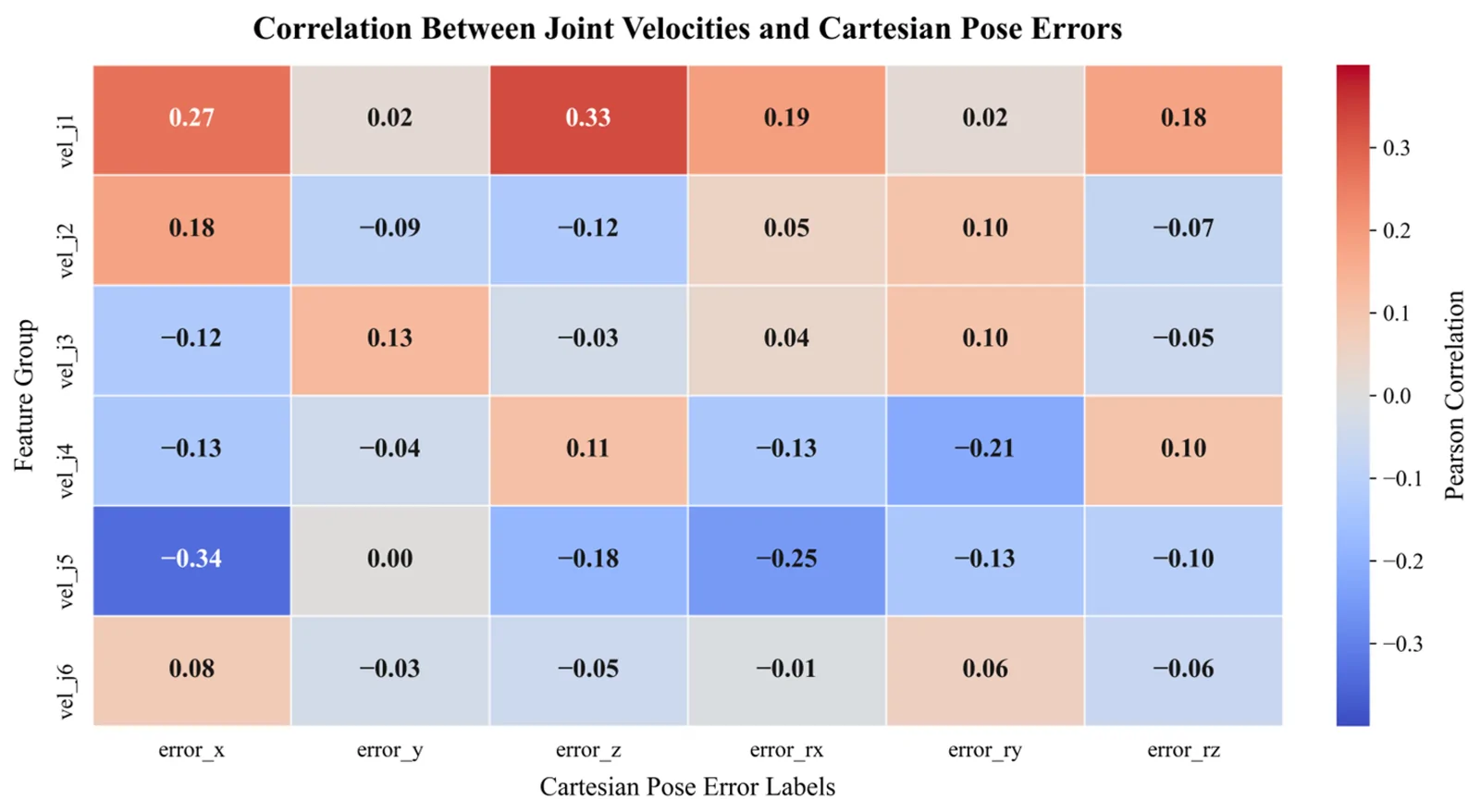
Heatmap showing the correlation between joint velocity and end-effector pose error (halfspeed;playload4.5bl).

**Figure 10:**
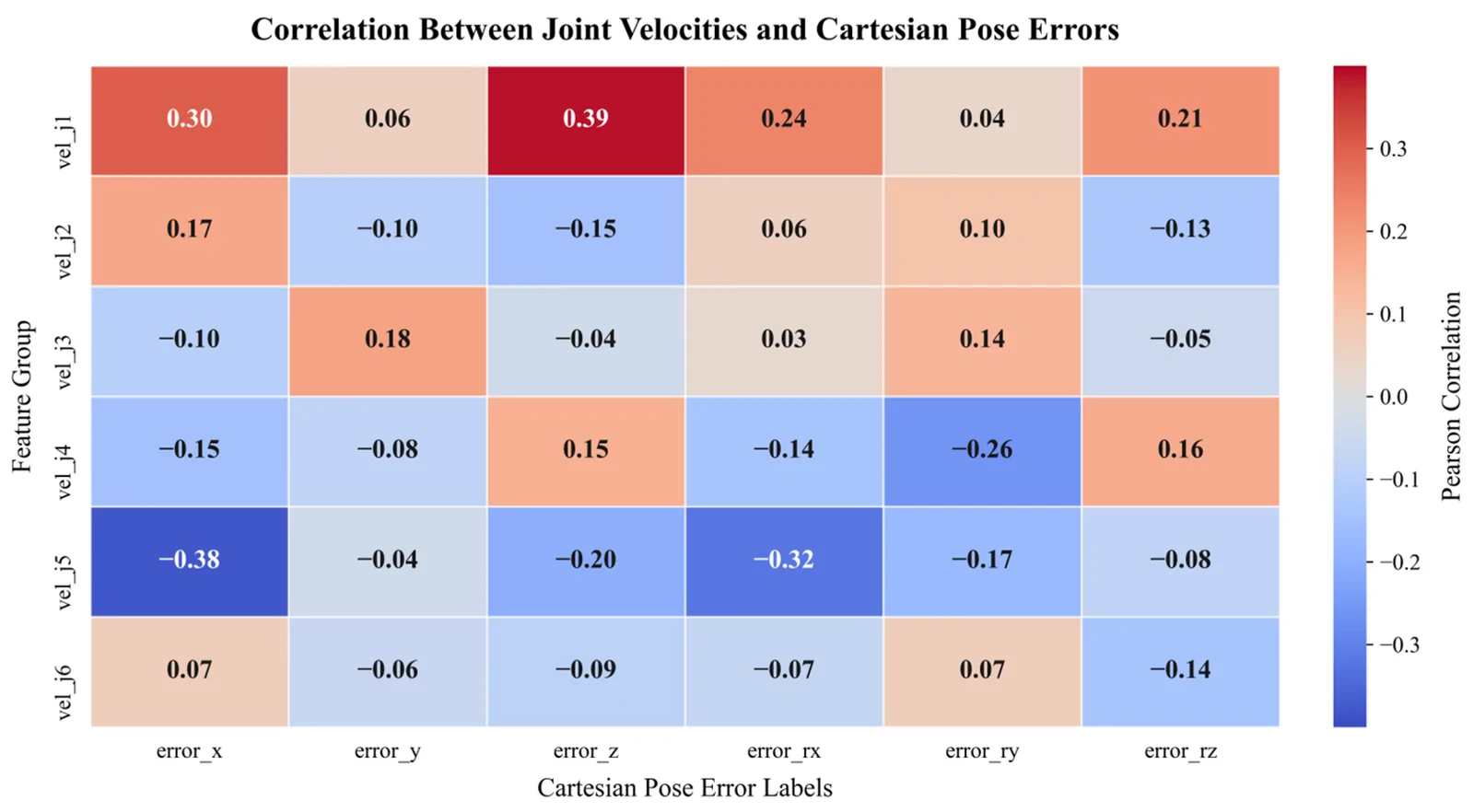
Heat map showing the correlation between joint velocity and end-effector position error (half-speed; payload 1.6bl).

As illustrated in Figure 9 and Figure 10, joint velocities also have a certain correlation with end-effector pose errors. Similar to joint currents, the velocities of different joints have uneven influences on various error dimensions. Among them, the velocities of Joint J1 and Joint J5 show relatively prominent correlations with error components. The correlation coefficients visualized in the heatmaps primarily reflect the linear correlation degree between variables. Nevertheless, the formation of robotic arm end-effector errors typically has two characteristics, which are nonlinearity and temporal coupling. Depending on this, correlation analysis alone cannot fully capture the evolving laws of error variation. Taking this limitation into consideration, this paper incorporates the Transformer encoder into the model architecture. Leveraging the model’s powerful capability to model temporal features and nonlinear mapping relationships, we achieve prediction and compensation for the pose errors of the robotic arm end effector.

#### 3.2.5. Typical Motion Curves of the UR5 Manipulator

To demonstrate the motion characteristics of the manipulator, this section takes the second joint (J2) of the UR5 as an example and presents its motion curves under typical operating conditions. The data are sourced from the NIST dataset, and we select a continuous 10 s operating segment under half-speed and 4.5 lb payload conditions, sampled at 125 Hz.

**Figure 11:**
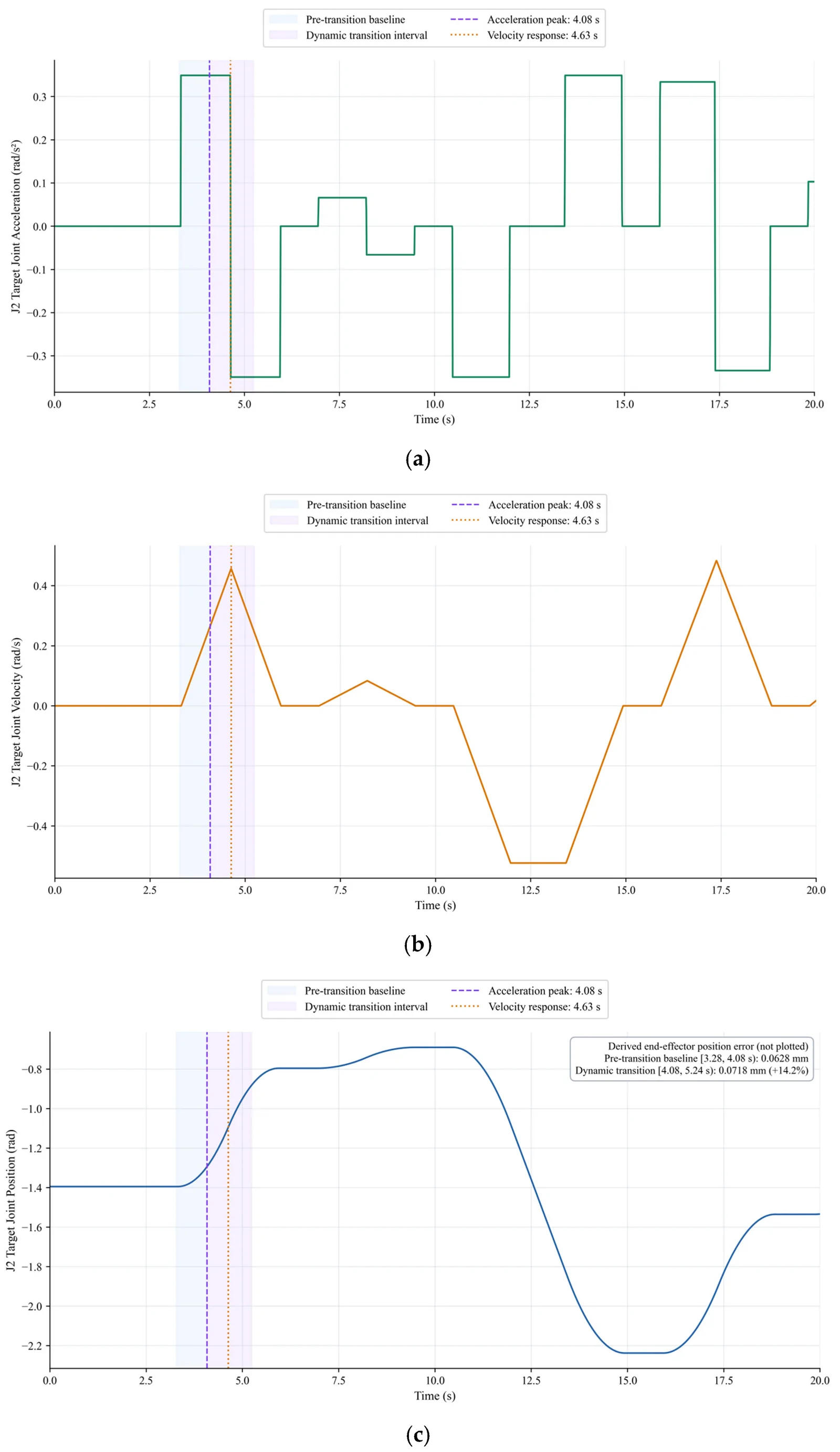
Motion curves of the second joint (J2) of the UR5 manipulator under typical operating conditions (half speed, 4.5 lb payload). (**a**) Joint angle of J2 versus time; (**b**) Joint velocity of J2 versus time; (**c**) Joint acceleration of J2 versus time.

As shown in Figure 11, the displacement (joint angle), velocity, and acceleration of the second joint (J2) exhibit distinct time-varying behavior throughout the motion trajectory. The joint angle curve in Figure 11a reflects the configuration variation in the shoulder joint as the manipulator moves between different target positions within the workspace. The velocity curve in Figure 11b reveals the dynamic adjustment of joint motion velocity; multiple acceleration and deceleration phases correspond to the start–stop transitions along the predefined trajectory. The acceleration curve in Figure 11c further shows the rate of change in velocity, which is closely related to the inertial forces and drive torques exerted on the joints.

It is worth noting that the end-effector errors of the manipulator depend not only on the current joint angle but also on historical velocity and acceleration states. As shown in the figure, the peak acceleration typically occurs before significant velocity fluctuations, and such dynamic transitions are often accompanied by increased position deviations of the end-effector. This observation strongly supports the validity of incorporating velocity and acceleration as input features into the Transformer-based error prediction model. Meanwhile, the temporal coupling among displacement, velocity, and acceleration within the historical time window further justifies the necessity of adopting sequence modeling to capture the error evolution pattern.

#### 3.2.6. Inverse Kinematics Solution and Validation

Inverse kinematics (IK) is the process of solving the joint angles given the target pose of the end-effector of a robotic manipulator. Compared with forward kinematics, the inverse problem is more challenging and faces the following three challenges: existence, uniqueness, and multiple solutions. For a 6-DOF serial manipulator such as the UR5, the main IK methods include analytical, numerical, and geometric approaches. The UR5 satisfies Pieper’s criterion (three adjacent joint axes intersect at a common point) [46], thus admitting analytical inverse kinematics solutions. It has been shown that there are eight sets of analytical solutions for the UR5. The optimal solution is selected from multiple candidates based on joint limit constraints and the minimum-travel criterion, i.e., minimizing the movement of each joint.

This paper adopts an analytical solution for the inverse kinematics of the UR5. This method is based on the modified D-H parameter model of the UR5 [41].

The solution procedure is as follows: first, we solve for the joint angle $q_{1}$ and compute the position $P_{5}^{0}$ of coordinate frame 5 relative to the base frame:

(24) $$P_{5}^{0}=T_{0}^{0}\left[\begin{array}{c} 0\\ 0\\ -d_{6}\\ 1 \end{array}\right]-\left[\begin{array}{c} 0\\ 0\\ 0\\ 1 \end{array}\right]$$

Then, we can get:

(25) $$q_{1}=\phi +\psi +\frac{\pi }{2},\psi =\arctan\left({\left(P_{5}^{0}\right)}_{y},{\left(P_{5}^{0}\right)}_{x}\right),\phi =\pm \arccos\left(\frac{d_{4}}{\sqrt{{\left(P_{5}^{0}\right)}_{x}^{2}+{\left(P_{5}^{0}\right)}_{y}^{2}}}\right)$$

$q_{1}$ has two solutions, corresponding to rightward or leftward rotation of joint 1.

Next, we solve the joint angle $q_{5}$. Given $q_{1}$, we compute $q_{5}$ using the position of frame 6 relative to frame 1:

(26) $$q_{5}=\pm \arccos\left(\frac{{\left(P_{6}^{1}\right)}_{z}-d_{4}}{d_{6}}\right)$$

$q_{5}$ also has two solutions, corresponding to upward or downward rotation of joint 5.

Finally, we solve the remaining joint angles. Once $q_{1}$ and $q_{5}$ are determined, the remaining joint angles ($q_{2},q_{3},q_{4},\;and\;q_{6}$) can be solved using algebraic methods or the Paden–Kahan subproblem approach [47], yielding up to eight sets of analytical inverse kinematics solutions. For practical feasibility, the optimal solution is selected based on joint limit constraints (the motion range of each joint of the UR5: $J_{1}:\pm 360^{\circ },J_{2}:\pm 360^{\circ },J_{3}:\pm 160^{\circ },J_{4}:\pm 360^{\circ },J_{5}:\pm 360^{\circ },\;and\;J_{6}:\pm 360^{\circ }$) and the minimum-travel criterion, i.e., minimizing the movement of each joint [48].

In the error compensation framework of this paper, inverse kinematics shoulders the following two key functions: (1) Error Label Validation: The error label validation is obtained from forward kinematics calculation (Equations (16) and (17)): $e_{t}=FK\left(q_{t}^{\mathrm{act}}\right)-FK\left(q_{t}^{\mathrm{tar}}\right)$. Inverse kinematics can be adopted to verify the self-consistency of the error label: the corrected end-effector pose $p_{t}^{\mathrm{tar}}+\Delta p$ is mapped back to the joint space via inverse kinematics, followed by a validity check to confirm whether the solved joint angles fall within the joint motion limits. (2) Compensation Command Generation: In practical industrial appliance, the final output of error compensation is the correction command of joint space. When model detects the end-effector error ${\hat{e}}_{t}$, the corrected end-effector pose $p_{t}^{\mathrm{tar}}+{\hat{e}}_{t}$ can be transferred to joint angle order $q_{t}^{\mathrm{comp}}$ via inverse kinematics and then sent to robot controller to perform. This process can be expressed as follows:

(27) $$q_{t}^{\mathrm{comp}}=IK\left(p_{t}^{\mathrm{tar}}+{\hat{e}}_{t}\right)$$

where $IK\left(\cdot \right)$ is the inverse kinematics function, the optimal solution that satisfies joint angle limits and minimum travel principle should be selected from multiple groups of solutions.

To verify the accuracy of inverse kinematics solutions, this paper conducts a forward and inverse kinematics consistency validation experiment. We randomly generate 1000 sets of joint angles $q_{\mathrm{rand}}$, and calculate corresponding end-effector pose $p=FK\left(q_{\mathrm{rand}}\right)$, then solve for joint angle $q_{\mathrm{ik}}$ from p via inverse kinematics, and compare the differences between $q_{\mathrm{rand}}$ and $q_{\mathrm{ik}}$, as shown in Table 6.

The above results demonstrate that the inverse kinematics solving method adopted in this paper achieves extremely high precision (pose error on the order of 10^−10^), which can provide a reliable bidirectional mapping between joint space and Cartesian space for subsequent error compensation. In terms of real-time performance, the analytic inverse kinematics solver takes approximately 169.864 microseconds (μs) per call, which is far shorter than the 8 ms (125 Hz) control cycle of the UR5 controller, thereby satisfying the real-time control requirements of industrial robots.

## 4. Results

### 4.1. Evaluation Metrics for Experimental Results

To comprehensively evaluate the performance of error prediction and compensation, this paper adopts mean absolute error (MAE), Root Mean Square Error (RMSE), and compensation improvement rate as core evaluation metrics. MAE represents the average absolute deviation between predicted values and ground-truth values, and its equation is given as follows:

(28) $$MAE=\frac{1}{n}\sum_{i=1}^{n}\left|\hat{y_{i}}-y_{i}\right|$$

Root Mean Square Error (RMSE) is more sensitive to large errors and can reflect whether the model has substantial local prediction bias. Its equation is shown as follows:

(29) $$RMSE=\sqrt{\frac{1}{n}\sum_{i=1}^{n}{\left(\hat{y_{i}}-y_{i}\right)}^{2}}$$

The compensation improvement rate (denoted as Improvement) measures the relative reduction ratio of errors after compensation compared with the uncompensated state, and it is defined as follows:

(30) $$\mathrm{Improvement}=\frac{{\mathrm{MAE}}_{\mathrm{before}}-{\mathrm{MAE}}_{\mathrm{after}}}{{\mathrm{MAE}}_{\mathrm{before}}}\times 100\%$$

### 4.2. Experimental Results and Analysis

It should be noted that all experiments in this paper adopt a strict historical lag window (the model takes features from time step t−L to t−1 as input to predict the pose error at time step t). All features including control current, driving torque and others are sampled from historical timestamps, and no data corresponding to the current time step t are incorporated. This design eliminates data leakage induced by synchronous coupling during model training and evaluation and guarantees the causal validity and engineering credibility of experimental results.

#### 4.2.1. Model Training Dynamics and Convergence Analysis

**Figure 12:**
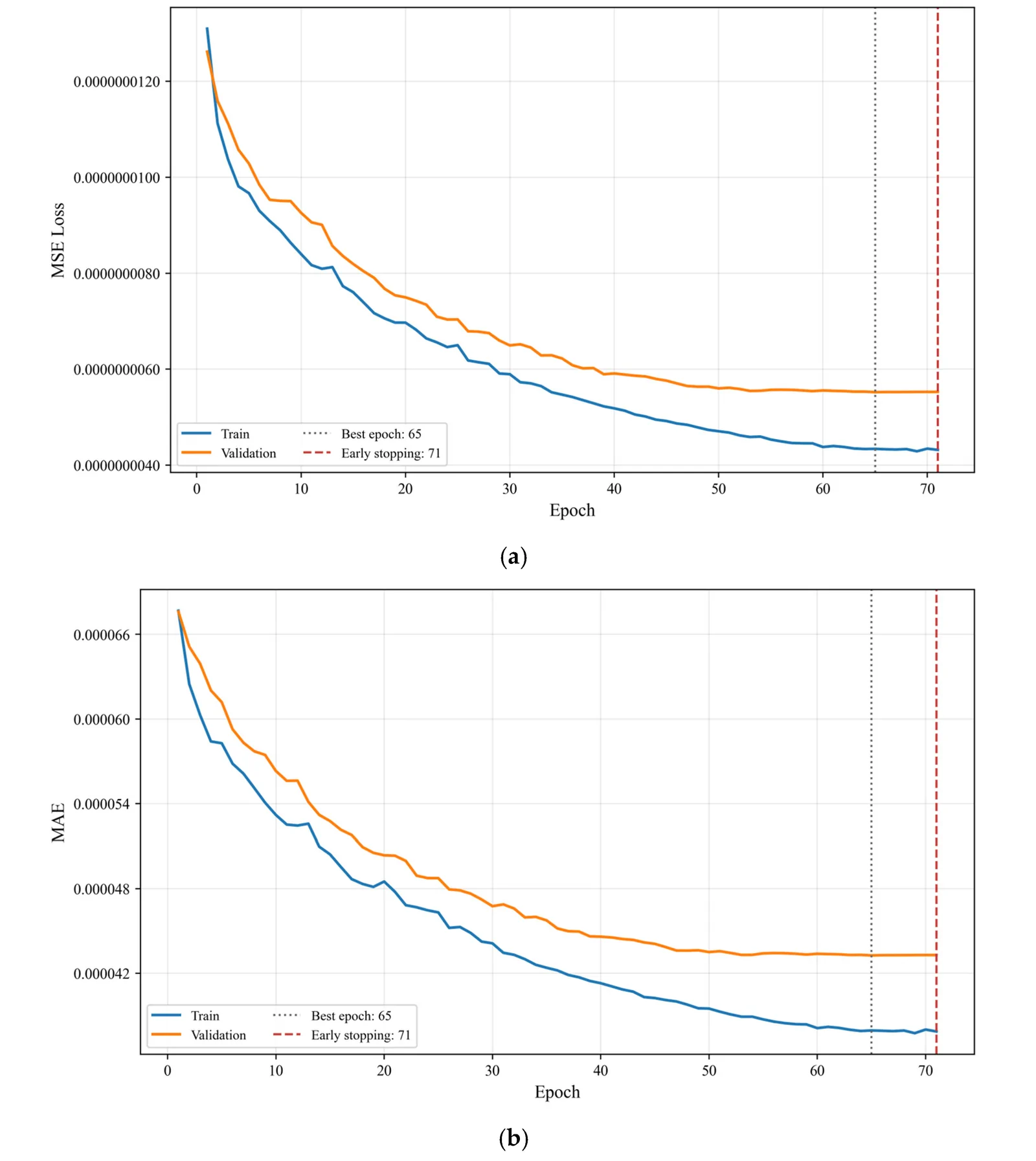

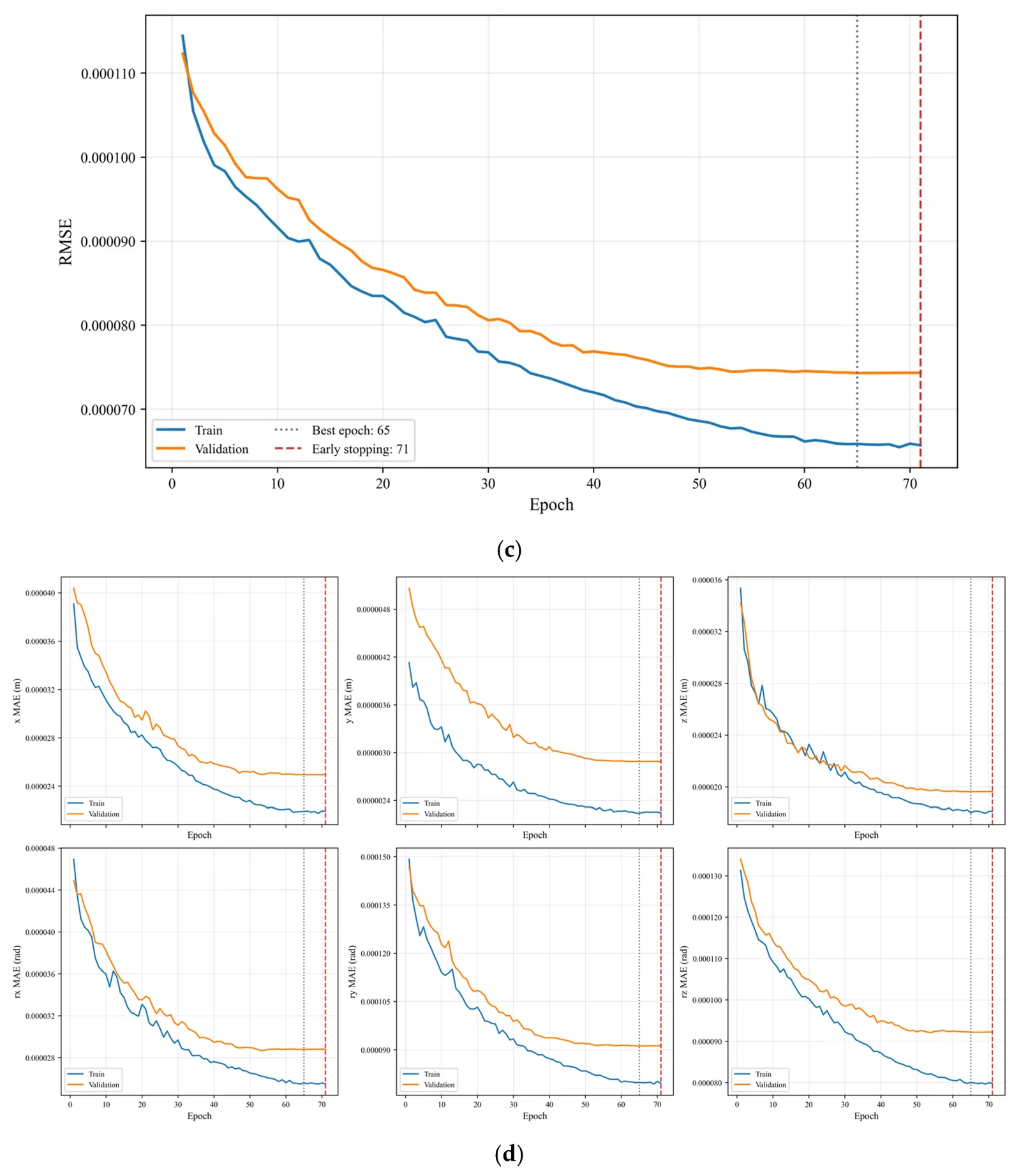
The dynamic curves based on Transformer error prediction model training. (**a**) MSE loss curves for the training and validation sets; (**b**) MAE curves for the training and validation sets; (**c**) RMSE curves for the training and validation sets; (**d**) MSE curves for the six output dimensions on the validation set.

In order to evaluate the training behavior and convergence features of the proposed Transformer error prediction model, this subsection shows the evolutionary trends of key performance indicators throughout the entire training process. Figure 12 illustrates the curves of MSE loss, MAE and RMSE on the training set and validation set varying with training epochs, as well as the MAE trajectories of each output dimension on the validation set.

As shown in Figure 12a, the MSE loss of the training set and validation set drop rapidly within the first 20 epochs, demonstrating the model can effectively capture the dominant error patterns from sequential input features. After around 40 epochs, the loss curve enters a plateau phase, with further marginal reduction in loss values. Throughout the entire training process, the validation set loss remains lower than training set loss, showing that the model does not suffer from sever overfitting. This phenomenon can be attributed to the Dropout regularization strategy adopted in Transformer encoder layers (dropout rate = 0.1).

The MAE and RMSE curves in Figure 12b,c exhibit a similar downward trend. The RMSE values remain higher than the MAE values. This is an expected outcome because the squared term in the RMSE calculation amplifies the contribution of larger errors. As training proceeds, the gap between MAE and RMSE gradually narrows, indicating the model gradually suppresses the occurrence of large prediction outliers.

Figure 12d shows the MAE trajectories of six output dimensions on the validation set. Among the six output dimensions, the MAE values of pose component (rx, ry, and rz) remain higher than position component throughout the entire training process, which corresponds to the observation that pose errors are harder to predict because of their stronger nonlinearity and the multi-joint coupling effect. The MAE values in z dimension are the lowest among all the dimensions, which corresponds to the result shown in Table 7 that this dimension achieves highest improvement rate (45.98%).

Early stopping is triggered when the validation loss fails to decrease significantly for six consecutive epochs. As is shown on the vertical dashed line in Figure 12, the optimal model parameters are saved at the 65th epoch (taking this experiment as an example). The final model achieves an MAE of 0.00004228 on the validation set and an RMSE of 0.00007155; these results will be used for the following performance compensation evaluation in Section 4.2.2.

Overall, the training dynamics demonstrate the Transformer model can converge stably and efficiently for the robotic manipulator pose error prediction task, without apparent signs of overfitting or underfitting. The continuous decline of MAE and RMSE throughout the entire training process verifies the effectiveness of the adopted training strategy, including the AdamW optimizer, physics-aware regularization term, and exponential error amplification preprocessing method.

#### 4.2.2. The Analysis of the Compensation Effect and Compensation Performance

In the previous subsection, the convergence behavior of the Transformer model during training was validated. This subsection further evaluates the actual compensation performance of the trained model on the test set.

To further analyze how distinct input features affect the performance of the Transformer-based error compensation model, this paper conducts comparative ablation experiments on feature selection controlled by the DROP hyperparameter. All experimental configurations including data partitioning strategy, time window length, model architecture and training scheme remain basically consistent across groups.

The DROP parameter governs whether each group of features is fed into the model input: a value of True denotes discarding the corresponding feature group, while False indicates retaining it. By incrementally incorporating target joint positions, target velocities, target accelerations, target torques, target motor currents and joint control currents into inputs, we can quantify the model’s capacity to exploit various physical information, which provides a solid basis for selecting the final feature combination.

During feature construction, actual joint positions are only adopted to compute error labels. Specifically, labels are defined as the deviation between actual joint positions and target joint positions. Actual joint positions are excluded from model inputs to avoid data leakage in the test phase.

The features retained in experiments are mainly derived from target trajectory commands and control-side variables, which correspond well to information that can be obtained in advance or output in real time by physical industrial controllers. Thus, the proposed scheme possesses strong practical engineering value. The DROP configurations and input feature dimensions of the four groups of experiments are summarized in Table 8. All features listed in the table are derived from the historical time window from t−L to t−1, i.e., the controller records from time step t−L to t−1. No feature values at the current time step t are strictly included. This configuration prevents data leakage caused by synchronous coupling when the model predicts the error at time step t.

From Table 8, it can be seen that in Experiment 1, only the target joint position was used as the input, with an input dimension of six, mainly to verify whether the model could learn the basic trend of errors when relying solely on the target pose instructions. Experiment 2 added the target joint velocity and target joint acceleration, raising the input dimension to 18, to supplement the motion state information. Experiment 3 further added the target joint torque and target joint current, raising the input dimension to 30, to introduce control information related to the driving load and joint force. Experiment 4 continued to add the joint control current on the basis of Experiment 3, raising the input dimension to 36, to examine the supplementary effect of the control execution quantity on error prediction.

The overall results of the four experiments are shown in Table 9. The average MAE before compensation for each group of experiments was all 0.00006916. The table mainly compared the average MAE after compensation, the overall improvement rate in joint space, R^2^, and the average improvement of the end position and end posture obtained by converting the forward kinematics of UR5.

As can be observed from Table 9, the overall compensation performance of the model gradually improves as input features are expanded from single target joint positions to multi-source features including motion states, target driving quantities and control currents.

When only target joint positions are utilized, the average MAE after compensation reaches 0.00005484, with an overall improvement rate of 20.70% in joint space. This reveals that target positions alone can partially reflect error patterns induced by trajectory configuration variations. Nevertheless, lacking velocity, acceleration and driving-related information, the model has limited capability to characterize dynamic errors and load-dependent errors.

After incorporating target joint velocities and target joint accelerations, the average MAE after compensation drops to 0.00004584, the overall joint-space improvement rate rises to 33.72%, and the coefficient of determination R^2^ increases from 0.4013 to 0.5684. This indicates that manipulator pose errors are closely correlated not only with current target joint positions but also with velocity and acceleration states during motion. Velocity and acceleration can reflect the dynamic variations in joint motion to a certain extent, enabling the Transformer to easily capture the evolution trend of errors along with motion states within the time window.

In Experiment 3, target joint torques and target motor currents are further supplemented as inputs. The post-compensation average MAE is further reduced to 0.00004480, the overall joint-space improvement rate reaches 35.23%, and R^2^ climbs to 0.5884. Compared with Experiment 2, the performance gain is relatively marginal, yet the average improvement of end-effector rotational errors rises from 41.70% to 42.48%. Such results demonstrate that target driving features can supplement partial information associated with driving loads, joint forces and motor output statuses. However, under the current dataset and model setup, their contribution to overall error prediction is weaker than motion state features such as velocity and acceleration.

Experiment 4, which integrates target motion states, target driving quantities and joint control currents as inputs, achieves the optimal performance among the four groups. Its average MAE after compensation decreases to 0.00004228, the overall joint-space improvement rate hits 38.86%, and R^2^ is elevated to 0.6414. The average improvement rates of translational and rotational end-effector errors reach 42.48% to 42.97%, respectively. This proves that control currents deliver effective information about control execution processes, helping the model further characterize error sources between actual joint motion and target motion, and exert a particularly prominent effect on reducing rotational errors.

From the perspective of feature selection, the experimental results show that relying solely on target joint positions can deliver limited compensation performance with insufficient interpretability for complex errors. Incorporating velocity and acceleration brings the most significant performance boost, which verifies that motion state features act as critical inputs for error prediction. Further introducing target torques, target motor currents and control currents allows the model to absorb additional information from the driving and control layers, yielding more stable compensation outcomes.

Therefore, the subsequent experiments in this paper adopt the 36-dimensional feature combination of target motion states + target driving quantities + control currents as the primary input features of the Transformer error compensation model. This combination avoids data leakage caused by introducing actual joint positions, while integrating trajectory commands, motion states and control execution information. It achieves a favorable balance between prediction accuracy and practical engineering feasibility.

**Figure 13:**
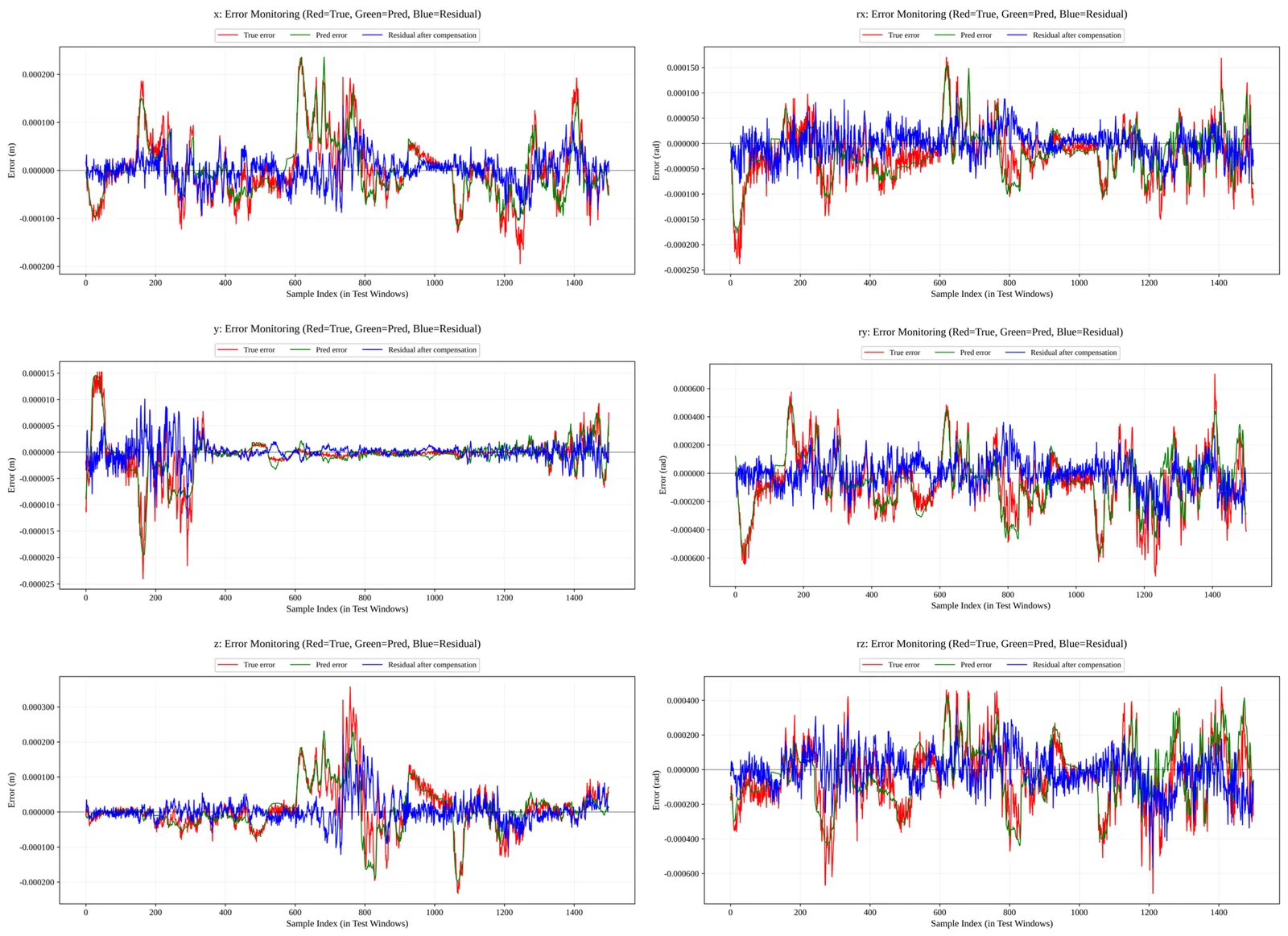
Compensation performance of manipulator across different dimensions.

The main experimental results are shown in Table 4 and Figure 13. After compensation by the Transformer model, the MAE of the last six dimensions decreased significantly compared to before compensation. The average MAE before compensation was 0.00013066, and after compensation, the average MAE decreased to 0.00008507, with an average improvement rate of approximately 34.89% across the six dimensions. This indicates that the model can effectively learn the mapping relationship between the operation characteristics of the robotic arm and the end error and can stably exert the compensatory effect in different output dimensions.

From the results of all dimensions, the z direction demonstrated the highest improvement rate which reached 45.98%, and the improvement rates on x and y directions were around 40%, while the orientation dimensions rx, ry, and rz all achieved a significant improvement. Due to the fact that position and orientation errors are varied in terms of dimension units and numerical range, directly comparing the absolute MAE on different dimensions is not completely equivalent, but the fact that the error decreases on the same dimension before and after the pose error compensation indeed proves that the model has learned the related error variation law.

Based on the above results, the DROP parameter comparison experiment verified the significant influence of feature selection on the error compensation effect of the robotic arm. The model performance improved with the increase in the amount of input feature information, indicating that the Transformer encoder can not only utilize the target trajectory information within the time window, but also extract dynamic patterns related to error changes from features such as speed, acceleration, torque, and current. Therefore, it is reasonable to introduce multi-dimensional control features from the control side into the error compensation model, and this also provides experimental basis for the subsequent analysis of compensation results in this paper.

#### 4.2.3. Compensation Performance Under Different Operating Conditions

The NIST dataset contains the following four typical operating conditions: light load and half speed (Condition 1), light load and full speed (Condition 2), heavy load and half speed (Condition 3), and heavy load and full speed (Condition 4). To evaluate the model’s robustness under various operating conditions, this paper classifies the test data by operating condition and evaluates the compensation performance of the Transformer model under each condition.

As shown in Table 10, the model achieves effective error compensation under all four working conditions, with improvement rates ranging from 38.86% to 52.17%. Notably, the pre-compensation MAE under full-velocity conditions (working condition 2 and working condition 4) is approximately 1.14 × 10^−3^, markedly higher than the pre-compensation MAE under half-velocity conditions (working condition 1 and working condition 3) ranging from 7 × 10^−5^ to 8 × 10^−5^, corresponding to a one-order-of-magnitude difference in error magnitude. This indicates that motion velocity acts as the dominant factor determining the magnitude of end-effector pose error. High-speed motion induces intensified dynamic effects, including elevated inertial force, aggravated joint flexible deformation and servo system tracking delay, which collectively lead to a remarkable rise in end-effector pose deviation.

Although the raw error under full-velocity working conditions is far larger than that under half-velocity working conditions, the model delivers more prominent compensation performance at full velocity as follows: the improvement rate reaches 52.06% for light load and full velocity (working condition 2) and 52.17% for heavy load and full velocity (working condition 4), both exceeding the improvement rates of 38.86% for light load and half velocity and 47.82% for heavy load and half velocity. This result reveals that error patterns are easier for the model to learn and capture when the error amplitude is large, enabling the model to eliminate a higher proportion of errors. In other words, larger error amplitudes provide stronger supervised signals, allowing the attention mechanism of Transformer to identify the mapping relationship between errors and input features more efficiently.

In terms of load conditions, the improvement rates under heavy-load scenarios (47.82% and 52.17%) are generally superior to those under light-load scenarios (38.86% and 52.06%), and the disparity is particularly obvious at half velocity (47.82% versus 38.86%, a gap of roughly 9 percentage points). It demonstrates that although increased end-effector load brings extra static deformation error, such error possesses favorable predictability. Flexible deformation caused by load follows consistent rules under identical load conditions, so the model can effectively learn and compensate for such systematic deviation.

In terms of residual error after compensation, the compensated MAE under the four working conditions converges to a similar order of magnitude (roughly 4.2 × 10^−5^~5.5 × 10^−5^). This phenomenon illustrates that the compensation capability of the model can reach a relatively unified lower precision limit regardless of initial error magnitude, reflecting favorable stability and consistency of the model in error correction.

Based on the above analysis, the following conclusions can be drawn: (1) velocity is the primary factor affecting error amplitude, and the error magnitude under full-speed working conditions is one order of magnitude larger than that under half-speed working conditions; (2) the model achieves higher improvement rates under large-error working conditions, verifying its stronger modeling capability for large-amplitude errors; (3) static deformation error induced by heavy load enjoys satisfactory predictability; and (4) the model reduces residual errors to comparable levels across diverse working conditions, demonstrating strong robustness and generalization performance. These conclusions offer vital references for the deployment of the model in practical industrial scenarios. Specifically, the proposed model can realize optimal compensation benefits for large-error working conditions such as high speed and heavy load.

To further evaluate the model’s robustness under diverse operating conditions, we divide the test data into four working-condition subsets according to load (1.6 lb and 4.5 lb) and moving speed (half velocity and full velocity) and evaluate the error compensation performance for each subset separately.

#### 4.2.4. The Test on the Generalization Ability of the Model

To evaluate the model’s generalization competence across various working conditions, this paper designs a Leave-One-Condition-Out verification experiment. Specifically, we use the data from three working conditions in sequence to train the model and perform testing on one remaining unseen working condition to test the compensation performance under the zero-transfer scenario. Meanwhile, to compute the performance gap, same-condition training (training and testing with data from an identical working condition) is performed for each working condition in this paper to serve as the upper bound of model performance.

As is shown in Table 11, the improvement rate of same-working condition in the table refers to the improvement rate acquired from the training and testing on target working condition (cited from Table 11); the improvement rate of cross-working condition refers to the improvement acquired from target working condition testing.

The performance gap is defined as the same-condition improvement rate minus the cross-condition improvement rate, reflecting the performance loss caused by cross-working condition generalization.

The model can achieve effective compensation in all cross-condition tests, with an improvement rate ranging from 17.74% to 32.07%.

However, the cross-condition generalization performance is significantly lower than same-condition; the gap is between 17.41% and 30.39%, indicating that the error modes across diverse working conditions have significant difference.

When working condition 4 (heavy load and full velocity) is adopted as the test set, the cross-condition improvement rate is merely 21.78%. The performance gap against the same-condition improvement rate (52.17%) reaches 30.39 percentage points, the largest among all working conditions. Working condition 3 (heavy load and half velocity) also suffers severe generalization difficulty with a performance gap of 30.08 percentage points and a cross-condition improvement rate of only 17.74%, the minimum value across the four groups. This phenomenon can be explained as follows: under heavy-load circumstances, joint flexible deformation and elastic effects are drastically intensified. These nonlinear physical effects present completely distinctive characteristic patterns under varying working conditions, making it difficult for the model to learn error characteristics under heavy loads from datasets collected under light-load conditions. Moreover, dynamic effects induced by full-speed operation (including increased inertial force and servo tracking delay) further raise the complexity of error patterns, rendering working condition 4 the most challenging target domain for generalization. In contrast, the cross-condition generalization performance is remarkably improved when the test working condition is under light load. Working condition 2 (light load and full velocity) achieves a cross-condition improvement rate of 32.07% with the performance gap narrowed to 19.99 percentage points. Working condition 1 (light load and half velocity) has a cross-condition improvement rate of 21.45% and the smallest performance gap of 17.41 percentage points in the four groups. Under light-load operation, the physical nonlinearity of the manipulator is weak, and joint flexibility and elastic deformation are insignificant. Errors mainly stem from geometric parameter deviation and kinematic errors, which maintain high similarity across different working conditions, thereby facilitating cross-condition transfer for the model. In terms of compensated MAE, working condition 4 exhibits the maximum residual error (0.00011980) when set as the test set, which is consistent with its maximum raw error (pre-compensation MAE = 0.0011449; see Table 8) and poor generalization performance. Working condition 1 obtains the minimum compensated MAE (0.00005696), which is close to its compensated result under the same working condition (0.00004228). Notably, even for working condition 3 (heavy load and half velocity) with the worst generalization capability, its cross-condition-compensated MAE (0.00006878) is notably lower than its pre-compensation original error (0.00008178), verifying that cross-condition transfer still achieves effective error reduction.

Above results demonstrate that the difference in error patterns across different working conditions is determined by load magnitude rather than velocity. The error source between heavy load and light load has the following fundamental discrepancy: flexible deformation induced by heavy loads constitutes an error mode absent under light-load working conditions. Therefore, a model trained under light-load conditions generalizes poorly to heavy-load scenarios. Based on this discovery, a working condition adaptive strategy is proposed for practical deployment as follows: (1) if the target working condition is light load, the common model can be used for direct deployment, the generalization performance is acceptable; (2) if the target working condition is heavy load, it is suggested to adopt a few target working condition to perform fine-tuning for the model to compensate the problem of insufficient generalization across various working conditions; and (3) for extreme circumstances like heavy load and full velocity, a special model is suggested to be adopted to ensure compensation precision.

#### 4.2.5. Prediction Error Distribution and Residual Analysis of the Proposed Method

**Figure 14:**
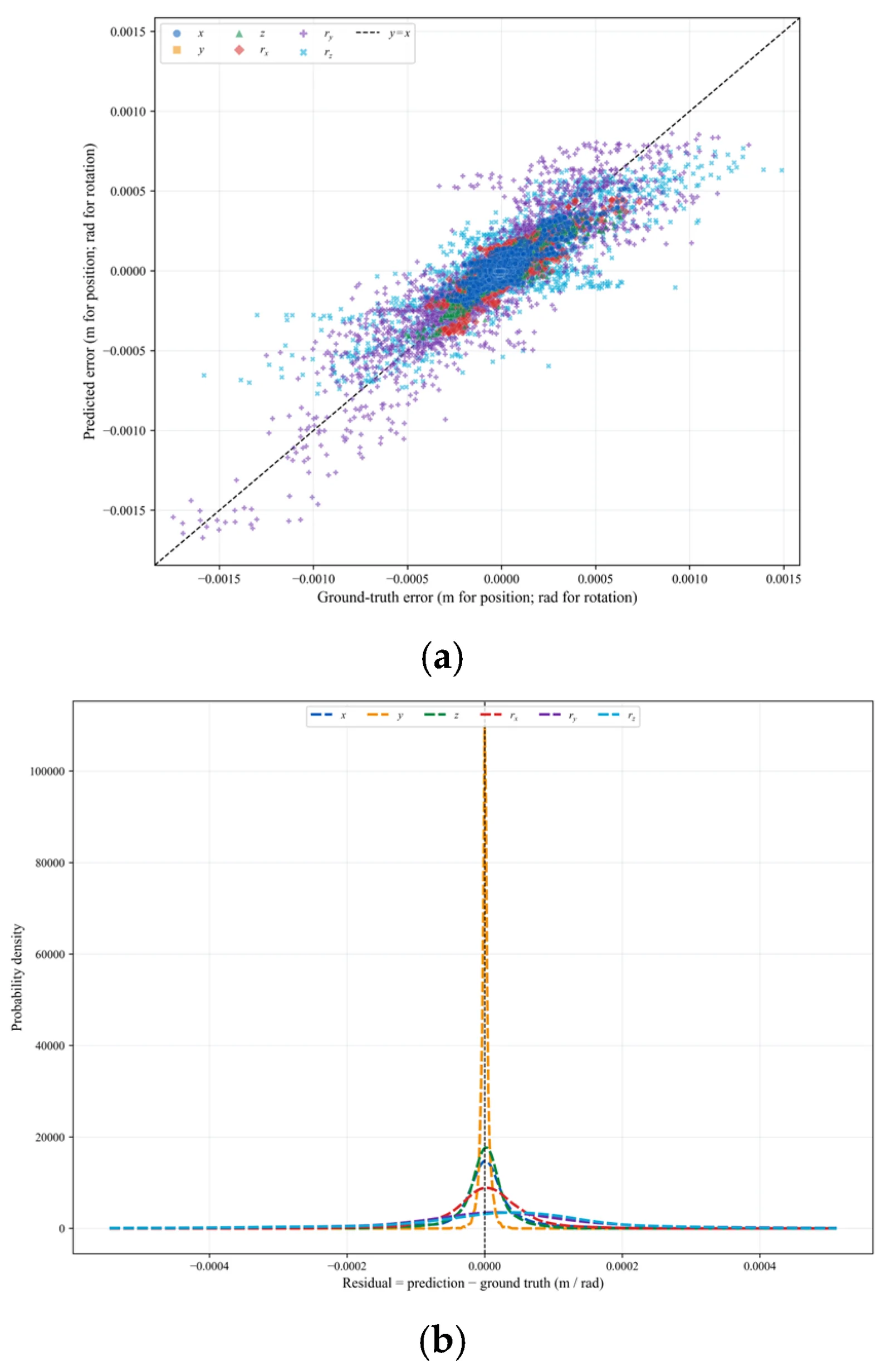

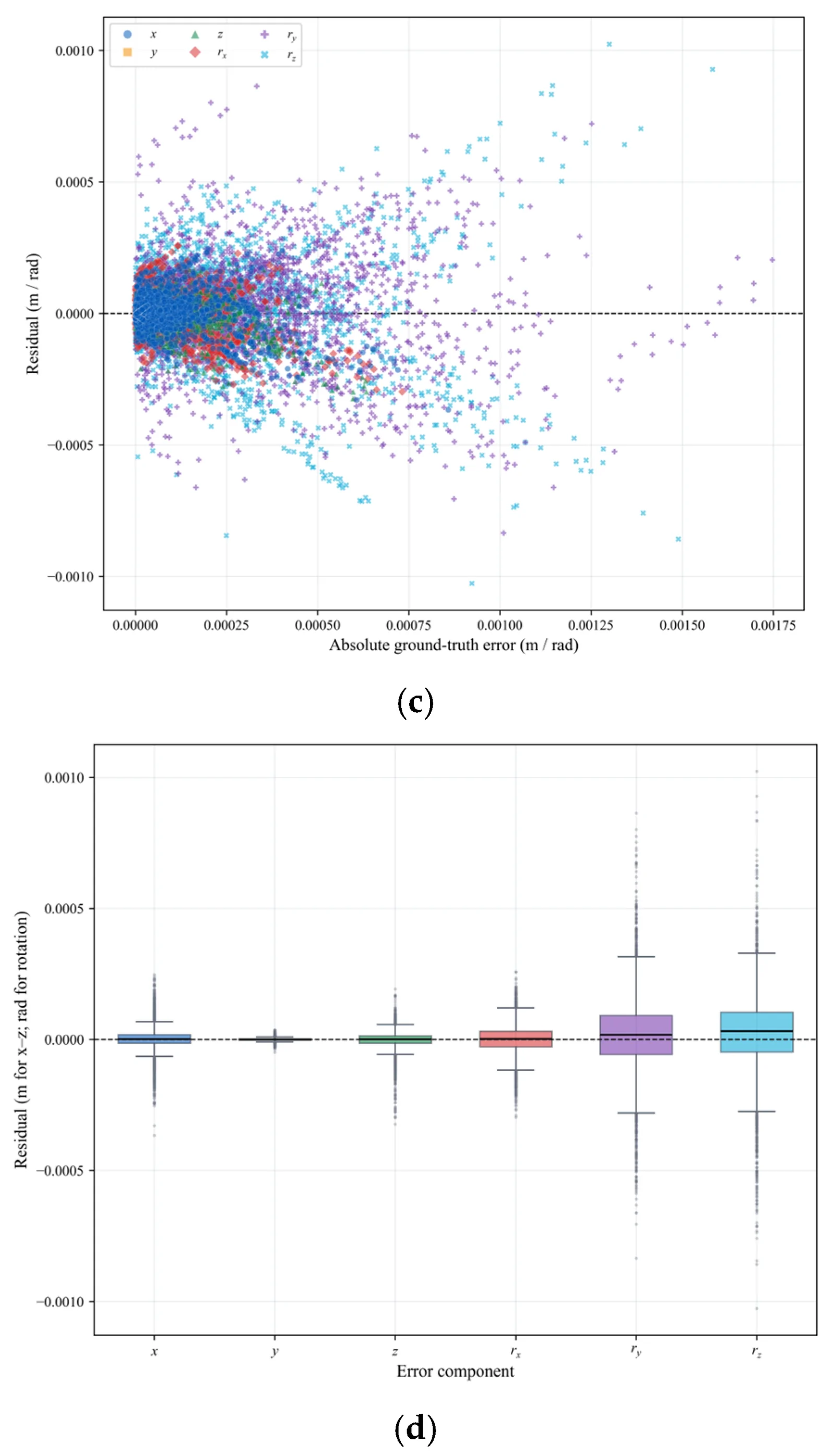
Prediction error distribution and residual analysis. (**a**) Scatter plot of predicted pose versus ground truth error; (**b**) Probability density distribution of prediction residuals; (**c**) Trend of prediction residuals versus the absolute value of ground truth error; (**d**) Box plots of residuals for each of the six output dimensions after compensation.

To further analyze the prediction behavior of the model, this paper performs residual analysis on the predicted errors. Figure 14a shows the scatter plot of predicted errors versus ground-truth errors. Under ideal circumstances, all data points should lie close to the diagonal line y=x. Most data points cluster near this diagonal, indicating good agreement between the model’s predictions and the ground-truth values. A few points deviating from the diagonal are mainly located in regions with relatively large errors, which is consistent with the observation in Table 4 that RMSE exceeds MAE. This indicates that the model exhibits some deviation in predicting large-error samples.

Figure 14b shows the probability density distribution of the residuals. The residual distribution approximately follows a normal distribution centered at zero, indicating no systematic overestimation or underestimation. The distribution has relatively high kurtosis, indicating that most prediction errors are concentrated near zero.

Figure 14c shows the residuals plotted against the absolute ground-truth error. For small errors, the residuals fluctuate randomly around zero without any clear trend. For larger errors, the residual dispersion increases, indicating that the model’s prediction certainty slightly decreases for large-magnitude errors. This may be attributed to the relatively limited number of large-error samples in the training set.

Figure 14d presents the boxplots of residuals for the six output dimensions. The residual medians for the translational components (x, y, and z) are closer to zero with smaller dispersion, whereas the orientation components (rx, ry, and rz) exhibit larger dispersion—particularly for ry and rz. This is consistent with the lower improvement rates for orientation components reported in Table 4, further confirming that orientation errors are more difficult to predict due to their stronger nonlinearity and multi-joint coupling effects.

#### 4.2.6. The Sensitivity Analysis of Hyperparameters

To investigate the model’s sensitivity to hyperparameters, this paper conducts parameter sweeping experiments for the time window length T, the number of attention heads h, and the number of Transformer encoder layers L, keeping all other parameters unchanged. The results are shown in Table 12.

Time window length T: When T increases from 20 to 100, the model performance improves continuously, with the improvement rate increasing from 34.05% to 38.15%. This indicates that a longer historical window provides more sequential contextual information, helping the model capture the cumulative effects of errors and long-term dependencies. When T is further increased to 200, the performance drops slightly (the improvement rate declines to 37.33%). This may be because an excessively long window introduces redundant information or noise, thereby increasing the learning difficulty. Therefore, the optimal window length is set to 100.

Number of attention heads h: When h increases from 2 to 4, the performance improves significantly, with the improvement rate rising from 37.17% to 39.26%, indicating that the multi-head attention mechanism can capture diverse correlations among input features from different subspaces. When h is further increased to 8, the performance drops slightly (38.15%), but when h is increased to 16, the performance somewhat improves (39.06%). Excessive attention heads may lead to parameter redundancy and overfitting. This paper selects four as the optimal number of attention heads after balancing computational efficiency and performance.

Number of encoder layers L: When L increases from 2 to 4, the performance improves notably, with the improvement rate increasing from 36.98% to 39.26%, demonstrating that a deeper network can extract higher-level abstract features. Nevertheless, when L is further increased to 6 and 8, the performance declines to 38.21% and 37.87%, respectively, indicating that an overly deep network may lead to overfitting or gradient vanishing problems in this task. Therefore, the optimal number of encoder layers is four.

Overall, the model can achieve an improvement rate of over 30% across a relatively broad hyperparameter range, demonstrating that the proposed method is relatively robust to hyperparameter choices, facilitating deployment and tuning across different application scenarios.

### 4.3. Comparative Experiment

#### 4.3.1. Selection of Comparative Experiment Method

To verify the effectiveness of the Transformer model, this paper selects the multi-layer perceptron (MLP) model as the main control experiment. In Galan-Uribe’s research, it was pointed out that the multi-layer perceptron has the advantages of easy embedded implementation, short training time, and the ability to provide high-quality nonlinear fitting models. Therefore, MLP is used as the core classifier and evaluator in this paper [49]. In addition, this paper also selects the tree model as a reference. Yang proposed a machine learning prediction model Global Configuration-Error Forest (GCE-Forest) to predict and compensate the trajectory errors of industrial robots based on digital twins [22]. At the same time, according to Messaoudi’s article, tree models are good at handling high-dimensional flat features and are suitable for this research task [50].

In the comparative experiment, the MLP model in this paper adopts the same data processing flow and input features as the Transformer model, ensuring the rigor of the comparative experiment. The difference lies in the input of the MLP model, which is to directly expand the multi-dimensional features within the time window and then input them into the fully connected network. Through multiple layers of nonlinear mapping, the six-dimensional error prediction results are output [51]. This setting can compare the differences between explicit temporal modeling and window expansion static modeling in the mechanical arm error compensation task.

#### 4.3.2. Design of Comparative Experiment

The design of the comparative experiment first needs to ensure that it can objectively reflect the performance differences in different models in this task. In the design of the comparative experiment, this paper first ensures the consistency of the dataset source, data preprocessing method, label construction, and evaluation index design. Therefore, all experiments in this paper are based on the same dataset, and the prediction target and evaluation method of the experiments are also consistent. The core idea of the experiment is to first predict the end-effector position error at the current time by the model and then use the predicted error for compensation of the target position, thereby comparing the changes in residual errors before and after compensation.

All experiments in this paper first clean and extract the original data and use the forward kinematics method to convert the joint space data into end-effector position data. Then, the error between the actual end-effector position and the target end-effector position is calculated as the supervision learning label. To reduce the influence of different dimensions and numerical ranges on model training, the input features and output errors are standardized in the experiments. For deep learning models, the standardization parameters are only fitted by the training set and applied to the validation set and test set to avoid test information leakage.

When constructing samples, both the Transformer and MLP experiments organize data in the form of time windows. Let the time window length be L; then, the input of the t-th sample can be represented as follows:

(31) $$X_{t}=\left[x_{t-L+1},x_{t-L+2},\ldots ,x_{t}\right]$$

Here, $x_{t}$ represents the operating state feature of the robotic arm at the t-th moment, and the corresponding label is the six-dimensional end-effector position and orientation error at the current moment:

(32) $$y_{t}=p_{t}^{actual}-p_{t}^{target}$$

This construction method enables the model to predict the current error based on the motion states within a certain period prior to the current moment. The key difference is that the Transformer retains the temporal structure of the input sequence, while the MLP flattens the features within the window and performs regression prediction.

Due to the strict time series characteristics of the robotic arm operation data, this paper adopts the method of dividing the training set, validation set, and test set in chronological order, without random shuffling. The training set is used for model parameter learning, the validation set is used for hyperparameter adjustment and early stopping judgment, and the test set is only used for the final evaluation of the compensation effect. This approach can more closely simulate the situation of using historical data to predict subsequent operation errors in practical applications, while avoiding the leakage of temporal information caused by random division.

For evaluation, this paper adopts the MAE before compensation, the MAE after compensation, and the average improvement rate as the primary comparison metrics, with the results reported separately for the six error dimensions. By maintaining the consistency of the experimental process, the comparison results of different models can be made more fair, thereby providing a basis for analyzing the effectiveness of the Transformer model in the task of robot arm pose error compensation.

#### 4.3.3. Comparison Experiment Results and Analysis

The experiment results showed that the MLP model has a certain level of compensation capability, which means the input features chosen by the paper contain information related to terminal errors, and the data-driven compensation logic is reasonable. By comparison, the overall improvement rate of MLP reaches 16.71%, as shown in Table 13. The improvement rate of the Transformer model was about 39.93%, as shown in Table 14. It is clear that the compensation effect of the MLP cannot compete with the Transformer model. This shows that only feeding flattened time window features into the network can hardly make full use of the internal temporal order and context of the sequence. However, the Transformer can more effectively model the impact of historical states on the current error and thus achieve a better compensation effect for this task.

In addition to the above comparative experiments on MLP, supplementary analysis of two tree-based models, XGBoost and Random Forest, is carried out in this paper, as shown in Table 14.

The tree-model experiments adopt the same residual learning framework as the error compensation task: first, the six-dimensional residual between the target end-effector pose and the actual end-effector pose is calculated. The residuals are scaled up by a factor of 1000 to optimize the numerical magnitude distribution, followed by standardization via StandardScaler. Afterwards, PyBullet is utilized to solve inverse kinematics and extract 36 inverse kinematics (IK) related input features. Finally, 5-fold cross-validation is applied to train and store the models.

These tree-based models are not selected as the primary model of this paper. Instead, they serve as comparative baselines to investigate the performance of traditional ensemble learning algorithms for manipulator pose error compensation.

### 4.4. Ablation Experiment

To verify the respective effectiveness and synergistic benefit of physical consistency restraint loss function and non-linear exponential error amplification strategy, this paper designs four independent experiments. All the experiments adopt identical data distribution scheme (training set 70%, validation set 15%, and testing set 15%), identical time window length (L = 100) and identical training hyper-parameters (AdamW optimizer, learning rate of 2 × 10^−4^, batch size 256, maximum training epoch of 100, and early-stopping mechanism) to ensure the comparability of each experiment result.

As shown in Table 15, the specific configurations of four experiments are listed below: experiment A (baseline model): it adopts standard Transformer encoder and regression head architecture and applies conventional MSE loss function to carry out training. Neither physical constraint loss nor exponential amplification for error labels is adopted. This group serves as a benchmark for performance comparison and is used to measure the independent contribution and cumulative gain of the two proposed strategies.

Experiment B (+physical constraint loss): On the basis of baseline model, the physical consistency loss function (Equation (11)) is introduced with weight coefficient λp = 0.3. The loss function is composed of the weight sum of MSE term and physical regularization term. This group does not adopt exponential error amplification so as to independently verify the improvement of prediction precision brought by physical constraints.

Experiment C (+exponential error amplification): Based on the baseline model, nonlinear exponential amplification is performed on error labels (Equation (19), α = 5.0). After model prediction output, inverse logarithmic transformation (Equation (20)) is applied to restore outputs to the original physical scale. The model is trained by MSE loss function without physical constraints. This group is used to independently verify the improvement effects of exponential amplification on convergence velocity and prediction precision.

Experiment D (Full Model): The physical consistency constraint loss ($\lambda_{p}=0.3$) and exponential error amplification (α = 5.0) are adopted simultaneously, constituting the complete physics-aware Transformer framework proposed in this paper. This experimental group is designed to verify the synergistic gain and complementarity of the two strategies. For each experimental group, the optimal model parameters are saved on the validation set, and the compensation performance is evaluated on the identical test set. The average post-compensation MAE, improvement rate, and epochs required for convergence of each model are recorded to calculate the individual contribution and superposition gain of each strategy.

All experiments are conducted with identical data partitioning, time window (L=100), and training hyperparameters. The improvement rate is calculated relative to Experiment A (baseline). MAE denotes the six-dimensional mean absolute error. “Epochs required for convergence” refers to the training epoch when the validation set MAE reaches its minimum value. The ablation experiment results are presented in Table 16.

From the ablation experimental results, the following conclusions can be drawn: (1) the physical constraint loss is effective when used alone (A vs. B): after introducing the physical constraint, the improvement rate rises from 27.39% to 31.83%, with an absolute increment of 4.44 percentage points. This verifies that the physical consistency constraint can improve prediction accuracy by leveraging dynamic prior knowledge. (2) Exponential error amplification achieves standalone effectiveness (A vs. C): the improvement rate increases from 27.39% to 33.65% after the introduction of exponential amplification, corresponding to an absolute gain of 6.26 percentage points, which outperforms the improvement brought by the physical constraint. More importantly, the convergence speed is improved by approximately 35% (the convergence epoch drops from 95 to 62), demonstrating that exponential amplification effectively alleviates the gradient vanishing issue caused by minor errors. (3) The two strategies deliver complementary gains (D vs. B/C): the full model achieves an improvement rate of 39.93%. Compared with the model using only physical constraints (31.83%) and the model using only exponential amplification (33.65%), the full model gains an additional 8.10 and 6.28 percentage points respectively. The combined gain of the two strategies (12.54%) exceeds the sum of their individual contributions (4.44% + 6.26% = 10.70%), which proves the orthogonality and synergistic effect of the two mechanisms. Specifically, the physical constraint provides physically reasonable guidance for the prediction direction, while exponential error amplification strengthens gradient signals from the perspective of numerical sensitivity.

## 5. Discussion

### 5.1. Work Summary and Limitation Analysis

This paper conducts research on end-effector pose error prediction and compensation for the UR5 manipulator. Aiming at the temporal, nonlinear and multi-factor coupling characteristics of manipulator errors, a Transformer encoder-based error prediction and compensation method is proposed in this work.

First, this paper analyzes the background and significance of accuracy improvement for industrial robots and reviews existing research on traditional error compensation methods, machine learning algorithms, and deep learning temporal sequence modeling approaches. Next, data preprocessing, including data cleaning, feature selection, forward kinematics transformation and six-dimensional end-effector error label construction, is completed based on the public NIST dataset. In terms of model design, a Transformer regression model is constructed, which takes multi-dimensional controller feature time windows as input and outputs six-dimensional end-effector pose errors. For experimental evaluation, MAE and compensation improvement rate are adopted to quantify model performance, with comparative experiments conducted against baseline models including MLP, XGBoost and random forest.

Experimental results demonstrate that the Transformer model can effectively reduce six-dimensional end-effector pose errors of the manipulator. The average MAE drops from 0.00006916 before compensation to 0.00004272 after compensation, corresponding to an average improvement rate of approximately 39.93%, which significantly outperforms baseline MLP and tree-based models. The average improvement rates of XGBoost and random forest in supplementary experiments range from 17.44% to 17.88%. This indicates that tree models can fit partial residual error patterns, yet their overall compensation performance is inferior to the Transformer. Such results verify that mining multi-feature state information within historical time windows via the self-attention mechanism can well capture temporal dependencies and nonlinear coupling relationships hidden in manipulator error variations. Furthermore, this paper designs an intelligent manipulator error compensation system and elaborates its implementation scheme covering system requirements, overall architecture, functional modules and operation workflows.

Nevertheless, several limitations remain in this research. First, all experiments are offline validations based on public datasets; online compensation tests on a physical UR5 manipulator platform have not been carried out. Therefore, the model’s stability and latency impact under real-time control environments require further verification. Second, rotation vector differences are adopted as an approximate representation of pose errors in this paper. Though this scheme facilitates implementation and alignment with controller data, more rigorous metrics can be adopted to quantify attitude errors within the strict three-dimensional rotation space.

Regarding future system deployment, despite the outstanding compensation accuracy of the Transformer model, real-world industrial robot control systems impose stringent requirements on real-time performance, stability and maintainability. A two-stage deployment strategy is proposed for subsequent research as follows: in the offline stage, the Transformer model is utilized to complete high-precision error modeling and parameter optimization; in the online or quasi-online stage, lightweight Transformers, knowledge-distilled student models or tree-based models are selected to rapidly predict compensation values according to available computing resources. Random forest or XGBoost can serve as lightweight fast compensation modules for working conditions with low precision requirements yet high cycle time demands. For scenarios requiring superior trajectory stability and handling complex dynamic errors, temporal deep learning models are prioritized. Such hierarchical deployment architecture enables the proposed compensation system to better match the practical demands of industrial applications.

In the model input design, this paper strictly adheres to the principle of temporal causality, adopting a purely historical lag window (features from time steps t−L to t−1 are used to predict the error at time t). This completely eliminates data leakage that could arise from synchronous coupling between variables such as control current and drive torque and the current tracking error. Although this causal constraint slightly reduces the theoretical upper bound of model performance (the improvement rate decreases by approximately 2–3 percentage points compared with a non-causal setup that uses features from time step t), it ensures the model’s effectiveness in real industrial online prediction scenarios—in practice, the controller cannot know the current error before it occurs, making predictions based on contemporaneous features physically infeasible. We choose to sacrifice a small amount of performance in exchange for causal soundness and engineering practicality, a design philosophy that holds significant value for the reliable deployment of industrial-grade error compensation systems.

It should be emphasized that the Transformer encoder adopted in this paper is inherently a standard architecture and therefore cannot be considered the core methodological innovation. The real contribution of this paper lies in constructing a physics-aware error prediction framework that takes the standard Transformer as the base regression model and combines the following two key strategies: the physical consistency constraint loss and the exponential error amplification strategy. More importantly, the two proposed strategies are not dependent on the specific architecture of the Transformer and exhibit strong transferability: both the physical consistency constraint loss and the exponential error amplification strategy can be applied to other regression models such as MLP and LSTM, offering generalizable methodological insights for a broader range of robotic manipulator error compensation studies.

### 5.2. Challenges and Prospects for Online Deployment

Based on the results and analysis of the above experiments, this section further discusses the limitations of the proposed method and future research directions, focusing on the critical challenges encountered in transitioning from offline verification to online deployment.

Although this paper has verified the effectiveness of the Transformer model for robotic manipulator pose prediction on public datasets, several key challenges remain prominent when extending it from offline prediction to online closed-loop compensation under practical industrial conditions. This section discusses the following four key aspects: computational latency, control cycle compatibility, closed-loop stability, and coordination between motion planning and error compensation.

(1) Computational Latency and Real-Time Inference

The adopted Transformer encoder consists of four encoder layers and eight attention heads, with approximately 8.5 million parameters. The inference latency on an NVIDIA A100 GPU is about 3–5 ms. Nevertheless, computational resources are often limited in practical industrial deployment, e.g., on embedded controllers or edge computing devices, which may lead to a significant increase in model inference latency. Previous studies have shown that the inference latency of a standard Transformer on CPU can reach 8.1 ms [52], while the control cycle of industrial robots is typically 8 ms (125 Hz) or even shorter. This means that the inference latency of a standard Transformer may approach or even exceed the control cycle under resource-limited conditions, thereby failing to meet real-time operational requirements. To address this issue, future work could explore model compression techniques such as knowledge distillation, parameter pruning, and quantization to reduce inference latency to within 3 ms. In addition, an event-triggered inference mechanism can be adopted, where model inference is triggered only when error accumulation exceeds a threshold or the motion state undergoes significant changes, rather than performing full inference on every control cycle, thereby reducing the computational load while ensuring compensation accuracy.

(2) Control Cycle Compatibility

The inference frequency of the Transformer model should be synchronized with the robot control cycle. The sampling frequency of the UR5 controller is 125 Hz (control period of 8 ms). If the model’s inference time exceeds the control cycle margin, it will delay the control commands and compromise real-time performance. A feasible solution is to adopt a pipelined parallel architecture, where model inference is decomposed into multiple computational stages, with only part of the computation completed within each control cycle, so that the inference result is output after several cycles, with a prediction–correction mechanism compensating for latency-induced errors. Moreover, drawing on the idea of pre-specified time control [53], a predefined convergence time can be set for error compensation to ensure that compensation values are computed and take effect within the specified time, achieving better synchronization with the control cycle.

(3) Closed-Loop Stability

In offline validation, error compensation is implemented in an open-loop feedforward manner as follows: the target pose is directly corrected after the model predicts the error, without closed-loop feedback. Nevertheless, during online deployment, the error compensation module needs to cooperate with the robot’s underlying servo control system to form a closed-loop system. At this point, the injection of compensation values may introduce additional phase lag or amplitude disturbance, which impairs closed-loop stability. Specifically, biased prediction errors from the model may generate erroneous compensation values. These mismatched corrections may cause conflicts between the controller outputs and the actual dynamic characteristics of the manipulator, potentially triggering oscillations or even system instability. To address this issue, we can draw on the event-triggered and hierarchical control ideas from distributed cooperative control [52]. An adaptive compensation threshold is configured at the control layer as follows: compensation is activated only when the confidence of the predicted error exceeds the preset threshold, while the original control command is retained otherwise. Meanwhile, a robust adaptive mechanism can be introduced to enable the compensation module to dynamically adjust the compensation gain according to real-time feedback signals, thereby guaranteeing closed-loop stability.

(4) Coordination between Motion Planning and Error Compensation

Error compensation should not be executed independently of motion planning. For practical industrial tasks, trajectory planning, velocity planning, and error compensation should be coordinated within a unified optimization framework. The superposition of compensation values may alter the end-effector trajectory, thereby compromising path smoothness and acceleration continuity. Incoordination between compensation commands and planned trajectories may lead to abrupt acceleration changes or torque overload, deteriorating machining quality and shortening equipment service life. Drawing on smooth path generation methods in motion planning research [54], future work could embed error compensation into the motion planning layer, where the influence of predicted errors is considered during trajectory generation to achieve coordinated optimization of trajectory planning and error compensation. Specifically, an error compensation term can be added to the planning objective function, so that the generated trajectory satisfies kinematic constraints while minimizing the adverse impact of predicted errors.

(5) Transition Route from Offline Validation to Online Deployment

Based on the above analysis, this paper proposes a progressive transition route from offline validation to online deployment:

Stage 1: Deploy a lightweight Transformer model on an industrial computer or edge server to provide feedforward error compensation references, with adoption decisions made by operators or a supervisory system.

Stage 2: Connect the compensation module to the robot control system for semi-closed-loop operation. Compensation values are released after manual confirmation or rule-based verification, gradually accumulating online operational data.

Stage 3: After fully verifying the stability and reliability of the compensation module, full closed-loop online compensation is realized. The model is continuously fine-tuned with online-collected data to adapt to error characteristics under specific operating conditions.

The resolution of the above challenges relies not only on model optimization but also on interdisciplinary collaboration among control theory, real-time systems, and robotics. This work establishes a preliminary data-driven foundation for future research in this direction. Future research will focus on lightweight model design, event-triggered inference mechanisms, and closed-loop stability verification.

## 6. Conclusions

This paper proposes a physics-aware Transformer-based framework for manipulator pose error prediction and compensation. The core contributions lie in (1) the integration of a physics-consistent constraint loss that enforces directional consistency between predicted errors and joint torque/end-effector velocity and (2) a nonlinear exponential error amplification strategy that enhances gradient signals for small-amplitude errors. Ablation studies confirm that each strategy independently contributes to performance improvement (4.44% and 6.26% respectively), and their combination yields a synergistic gain of 12.54% over the baseline. Taking multi-dimensional controller features within the historical time window as inputs, the model outputs the predicted six-dimensional end-effector pose error at the current timestep through linear projection, positional encoding, multi-layer self-attention encoders and a regression head. MAE, RMSE and compensation improvement rate are utilized to quantify the model performance. Comparative experiments against baseline models including MLP, XGBoost and random forest demonstrate that the proposed model achieves superior prediction accuracy and possesses broad application prospects in future industrial scenarios. Future work will focus on online deployment, including the following three aspects: (1) lightweighting the Transformer via knowledge distillation and model quantization to meet real-time control requirements; (2) designing an event-triggered inference mechanism to reduce inference frequency while maintaining compensation accuracy; and (3) performing closed-loop compensation experiments on a physical robot platform to verify the stability and robustness of the method.

## Figures and Tables

**Table 1:** Nomenclature.

Symbol	Description	Symbol	Descriptions	Symbol	Description
General Notation					
t	Time step index	L	Length of sliding time window	d	Input feature dimension per time step
$d_{k}$	Dimension of each attention head	$d_{model}$	Hidden dimension of the Transformer model	h	Number of attention heads
θ	Learnable parameters of the neural network				
**Kinematics Notation**					
$q_{t}^{tar}$	Target joint angles at time step t (6 × 1)	$q_{t}^{act}$	Actual joint angles at time step t (6 × 1)	$p_{t}^{tar}$	Target end-effector pose at time step t (6 × 1: x,y,z,rx,ry,rz)
$p_{t}^{act}$	Actual end-effector pose at time step t (6 × 1)	$e_{t}$	Six-dimensional end-effector pose error at time step t	$FK\left(\cdot \right)$	Forward kinematics function
$IK\left(\cdot \right)$	Inverse kinematics function	$T_{i}^{i-1}$	Homogeneous transformation matrix from frame i to frame i−1	$\alpha_{i-1},a_{i-1},d_{i},\theta_{i}$	Denavit–Hartenberg (D-H) parameters
$J_{1}∼J_{6}$	Joints 1 to 6 of the UR5 manipulator				
**Model Notation**					
$X_{t}$	Input feature matrix within the time window at time step t	${\hat{e}}_{t}$	Predicted six-dimensional pose error	Q,K,V	Query, key, and value matrices in attention mechanism
$W^{O}$	Output weight matrix of multi-head attention	PE	Positional encoding matrix	L	Total loss function
$\lambda_{p}$	Weighting coefficient for the physical constraint penalty	MSE	Mean squared error loss	MAE	Mean absolute error
RMSE	Root mean square error				
**Other Variables**					
α	Exponential amplification factor for error scaling				

**Table 2:** Transformer main structural parameters.

Parameter	Setting Value
Input Feature Dimension (per time step)	36
Length of Time Window L	100
Hidden Dimension $d_{model}$	128
Transformer Encoder Layers	4
Number of Attention Heads	8
Feed Forward Dimension	256
Dropout Ratio	0.1
Output Dimension	6 (x, y, z, rx, ry, rz)

**Table 3:** Modified D-H parameters for the UR5 manipulator.

Joint i	$\alpha_{i-1}$ (rad)	$a_{i-1}$ (m)	$d_{i}$ (m)	$\theta_{i}$ (rad)	Joint Range
1	0	0	0.089159	$\theta_{1}$	±360°
2	−π/2	0	0	$\theta_{2}$	±360°
3	0	−0.425	0	$\theta_{3}$	±160°
4	0	−0.39225	0.10915	$\theta_{4}$	±360°
5	−π/2	0	0.09465	$\theta_{5}$	±360°
6	π/2	0	0.0823	$\theta_{6}$	±360°

Note: $\alpha_{i-1}(rad)$: link twist angle (rotation angle from $z_{i-1}$ to $z_{i}$ about axis $x_{i-1}$). $a_{i-1}(m)$: link length (translational distance from $z_{i-1}$ to $z_{i}$ along axis $x_{i-1}$). $d_{i}$: link offset (translational distance from $x_{i-1}$ to $x_{i}$ along axis $z_{i}$). $\theta_{i}$: joint angle (rotational angle from $x_{i-1}$ to $x_{i}$ about axis $z_{i}$); all joints of the UR5 are revolute.

**Table 4:** Comparison reference of dataset content.

Number	Meaning	Types of the Data	The Number of the Data	The Size of the Character	Explanation
**A**	ROBOT_TIME	double	1	8	Time elapsed since the controller was started
**B-G**	ROBOT_TARGET_JOINT_POSITIONS	double	6	48	Target joint positions
**H-M**	ROBOT_ACTUAL_JOINT_POSITIONS	double	6	48	Actual joint positions
**N-S**	ROBOT_TARGET_JOINT_VELOCITIES	double	6	48	Target joint velocities
**T-Y**	ROBOT_ACTUAL_JOINT_VELOCITIES	double	6	48	Actual joint velocities
**Z-AE**	ROBOT_TARGET_JOINT_CURRENT	double	6	48	Target joint currents
**AF-AK**	ROBOT_ACTUAL_JOINT_CURRENT	double	6	48	Actual joint currents
**AL-AQ**	ROBOT_TARGET_JOINT_ACCELERATIONS	double	6	48	Target joint accelerations
**AR-AW**	ROBOT_TARGET_JOINT_TORQUES	double	6	48	Target joint torques
**AX-BC**	ROBOT_JOINT_CONTROL_CURRENT	double	6	48	Joint control currents
**BD-BI**	ROBOT_CARTESIAN_COORD_TOOL	double	6	48	Actual Cartesian coordinates of the tool: (x, y, z, rx, ry, rz), where rx, ry and rz is a rotation vector representation of the tool orientation
**BJ-BO**	ROBOT_TCP_FORCE	double	6	48	Generalized forces in the TCP
**BP-BU**	ROBOT_JOINT_TEMP	double	6	48	Temperature of each joint in degrees Celsius

**Table 5:** The impact of standardization on the experimental results.

	With Standardization	Without Standardization
**Overall Average Error Improvement**	49.21%	−215.67%

**Table 6:** Validation results of forward and inverse kinematics consistency.

Validation Metrics	Value	
Number of Testing Samples	100	
Success Solution Rate of Inverse Kinematics	100%	
Maximum Deviation of Joint Angle	$6.568\times 10^{-10}$ deg, ($1.146\times 10^{-11}$ rad)	
Average Deviation of Joint Angle	$5.382\times 10^{-12}$ deg, ($9.393\times 10^{-13}$ rad)	
Recompose Deviation of End-Effector Pose (Verified by Forward Kinematics)	Position Recompose Errors	Maximum $8.447\times 10^{-10}$ mm; Average $1.800\times 10^{-11}$ mm
	Pose Recompose Errors	Maximum $1.026\times 10^{-11}$ rad; Average $2.073\times 10^{-13}$ rad

**Table 7:** The compensatory effect of the Transformer model.

Dimension	MAE (Before Compensation)	MAE (After Compensation)	Improvement Rate
x	0.00004161	0.00002457	40.97%
y	0.00000463	0.00000288	37.78%
z	0.00003565	0.00001926	45.98%
rx	0.00004470	0.00002846	36.34%
ry	0.00015770	0.00009348	40.73%
rz	0.00013066	0.00008507	34.89%
Mean	0.00006916	0.00004228	38.86%

**Table 8:** Experimental settings for feature selection comparison.

Number of the Experiment	Characteristic Combination	DROP=False (Reserve)	DROP=True (Delete)	Input Dimension
Experiment 1	The object’s joint position only	target_state target_pos	Time target_actuation ctrl_curr joint_temp tool_pose tcp_force actual_feedback	6
Experiment 2	The moving condition of the object	target_state target_pos target_vel target_acc	Time target_actuation ctrl_curr joint_temp tool_pose tcp_force actual_feedback	18
Experiment 3	Target motion states + target driving quantities	target_state target_actuation	time,ctrl_curr joint_temp tool_pose tcp_force actual_feedback	30
Experiment 4	Target motion states + target driving quantities + control currents	target_state target_actuation ctrl_curr	time,joint_temp tool_pose tcp_force actual_feedback	36

**Table 9:** The comparison between different Transformer models under different characteristic combinations.

Number of the Experiment	Characteristic Combination	Input Dimension	The Average MAE After Compensation	R^2^	The Improvement Rate at the End	The Average Improvement Rate on End-Effector Position (x, y, z)	Average Improvement Rate on End-Effector Pose (rx, ry, rz)
Experiment 1	The object’s joint position only	6	0.00005484	0.4013	20.70%	24.26%	19.82%
Experiment 2	The moving condition of the object	18	0.00004584	0.5684	33.72%	41.70%	31.76%
Experiment 3	Target motion states + target driving quantities	30	0.00004480	0.5884	35.23%	42.48%	33.44%
Experiment 4	Target motion states + target driving quantities + control currents	36	0.00004228	0.6414	38.86%	42.97%	37.85%

**Table 10:** Comparison of compensation performance of Transformer model under diverse working conditions.

Working Condition	Load	Velocity	MAE Before Compensation	MAE After Compensation	Improvement Rate
Working Condition 1	1.6 lb	Half velocity	0.00006916	0.00004228	38.86%
Working Condition 2	1.6 lb	Full velocity	0.0011455	0.00005491	52.06%
Working Condition 3	4.5 lb	Half velocity	0.00008178	0.00004267	47.82%
Working Condition 4	4.5 lb	Full velocity	0.0011449	0.00005476	52.17%

**Table 11:** Evaluation of cross-working-condition generalization ability (Leave-One-Condition-Out validation).

Training Working Condition	Testing Working Condition	MAE of Testing Set After Compensation	Improvement Rate of Cross-Working Condition	Improvement Rate of Same-Working Condition	Performance Gap
Working Conditions 1, 2, 3	Working Condition 4 (heavy load and full velocity)	0.00011980	21.78%	52.17%	30.39%
Working Conditions 1, 2, 4	Working condition (heavy load and half velocity)	0.00006878	17.74%	47.82%	30.08%
Working Conditions 1, 3, 4	Working Condition 2 (light load and full velocity)	0.00007362	32.07%	52.06%	19.99%
Working Conditions 2, 3, 4	Working Condition 1 (light load and half velocity)	0.00005696	21.45%	38.86%	17.41%

**Table 12:** Effects of hyperparameters on model performance.

Parameter	Value	Validation Set MAE	Improve Rate	Optimal Value
Time Window T	20	0.00004560	34.05%	
	50	0.00004375	36.83%	
	100	0.00004277	38.15%	√
	200	0.00004288	37.33%	
Attention Head Number h	2	0.00004345	37.17%	
	4	0.00004201	39.26%	√
	8	0.00004277	38.15%	
	16	0.00004215	39.06%	
Encoder Layer L	2	0.00004358	36.98%	
	4	0.00004201	39.26%	√
	6	0.00004273	38.21%	
	8	0.00004297	37.87%	

**Table 13:** Results of compensation under MLP model.

Dimension	MAE (Before Compensation)	Average MAE (After Compensation)	Improvement Rate
x	0.00004113	0.00003774	8.23%
y	0.00000466	0.00000407	12.66%
z	0.00003530	0.00002216	37.23%
rx	0.00004532	0.00003996	11.82%
ry	0.00015735	0.00013109	16.69%
rz	0.00013108	0.00011320	13.64%
Average	0.00006914	0.00005804	16.71%

**Table 14:** Comparison between different experimental results under different models.

Model	Average MAE (Before Compensation)	Average MAE (After Compensation)	Improvement Rate
Transformer	0.00006916	0.00004272	39.93%
MLP	0.00006914	0.00005804	16.71%
XGBoost	0.00006395	0.00005280	17.44%
Random Forest	0.00006395	0.00005251	17.88%

**Table 15:** Experiment design table of ablation experiments.

Experiment	Model Configuration	Abbreviation
A	Standard Transformer + MSE Loss (Without Physical Constraints and Exponential Amplification)	Baseline Model
B	Transformer + Physical Constraint Loss ($\lambda_{p}=0.3$, Without Exponential Amplification)	+Physical Constraint Loss
C	Transformer + Exponential Error Amplification (α = 5.0, Without Physical Constraints)	+Exponential Amplification
D	Transformer + Physical Constraint + Exponential Amplification (Full Model)	Full Model

**Table 16:** Ablation experiment results: comparison between different configurations.

Experiment	Model Configuration	Average MAE (After Compensation)	Improvement Rate	Required Epoch for Convergence	Compared with Baseline Improvement
A	Full Model (Without Strategies)	0.00005023	27.39%	95	—
B	+Physical Constraint Loss	0.00004715	31.83%	88	+4.44%
C	+Exponential Amplification	0.00004589	33.65%	62	+6.26%
D	+Physical Constraint +Exponential Amplification (Full Model)	**0.00004272**	39.93%	78	+12.54%

## Data Availability

The original contributions presented in this study are included in the article. Further inquiries can be directed to the corresponding author.
